# Epilepsy with myoclonic-atonic seizures: genetic aetiologies, outcomes and prognostic indicators

**DOI:** 10.1093/braincomms/fcaf507

**Published:** 2025-12-29

**Authors:** Simona Pellacani, Simona Balestrini, Edoardo Fino, Carmen Barba, Mara Cavallin, Tiziana Pisano, Elena Parrini, Anna Rita Ferrari, Chiara Marzi, Laura Grisotto, Renzo Guerrini

**Affiliations:** Neuroscience and Human Genetics Department, Meyer Children's Hospital IRCCS, Florence 50139, Italy; Neuroscience, Pharmacology and Child Health Department, University of Florence, Florence 50139, Italy; Neuroscience and Human Genetics Department, Meyer Children's Hospital IRCCS, Florence 50139, Italy; Neuroscience, Pharmacology and Child Health Department, University of Florence, Florence 50139, Italy; Department of Clinical and Experimental Epilepsy, UCL Queen Square Institute of Neurology, WC1N 3BG London, UK; Neuroscience and Human Genetics Department, Meyer Children's Hospital IRCCS, Florence 50139, Italy; Neuroscience, Pharmacology and Child Health Department, University of Florence, Florence 50139, Italy; Neuroscience and Human Genetics Department, Meyer Children's Hospital IRCCS, Florence 50139, Italy; Neuroscience, Pharmacology and Child Health Department, University of Florence, Florence 50139, Italy; Neuroscience and Human Genetics Department, Meyer Children's Hospital IRCCS, Florence 50139, Italy; Neuroscience and Human Genetics Department, Meyer Children's Hospital IRCCS, Florence 50139, Italy; Neuroscience and Human Genetics Department, Meyer Children's Hospital IRCCS, Florence 50139, Italy; Department of Developmental Neuroscience, IRCCS Stella Maris Foundation, Pisa 56128, Italy; Department of Statistics, Computer Science, Applications, University of Florence, Florence 50139, Italy; Department of Environmental and Prevention Sciences, University of Ferrara, Ferrara 44122, Italy; Neuroscience and Human Genetics Department, Meyer Children's Hospital IRCCS, Florence 50139, Italy; Neuroscience, Pharmacology and Child Health Department, University of Florence, Florence 50139, Italy

**Keywords:** myoclonic, atonic, neurodevelopment, childhood epilepsy, drug resistance

## Abstract

Epilepsy with myoclonic-atonic seizures, formerly myoclonic-astatic epilepsy or Doose syndrome, accounts for 1–2.2% of childhood-onset epilepsies. We investigated genetic determinants, long-term clinical outcomes and prognostic indicators in a large cohort using homogeneous inclusion criteria. We studied 60 patients (26.7% female), mean age 14.5 years (±9.1, range 3.2–41), followed between 1986 and 2024 at two paediatric neurology centres. Average follow-up was 11.7 years. Inclusion criteria were seizure onset between 6 months and 8 years, generalized 2–6 Hz spike-wave discharges and video-EEG documented myoclonic-atonic, myoclonic seizures or both. We analysed clinical, EEG, neuroimaging, neuropsychological and genetic data obtained with next-generation sequencing. We used χ² test, *t-*test, Log-rank test, Cox regression, population-averaged logistic models and Benjamini–Yekutieli procedure to identify predictors of seizure outcome, intellectual disability and other neurodevelopmental comorbidities. We observed myoclonic-atonic seizures in 55/60 (91.7%), tonic-vibratory seizures in 44/60 (73.4%), absence seizures in 30/60 (50%), myoclonic seizures without post-myoclonic atonia in 25/60 (42%) and non-convulsive status epilepticus in 13/60 (21.7%). A ‘stormy’ onset occurred in 26/60 patients (43.3%). The most effective drugs were valproate, ethosuximide, benzodiazepines and phenobarbital, used in different combinations, whereas the newer drugs offered no benefit. Long-term outcomes were variable. Thirty-seven patients (61.7%) achieved seizure freedom after 5.1 years on average. We observed drug resistance in 23/60 patients (38.3%) and intellectual disability in 35/60 (58.3%). One adult patient died (mortality rate 1.80/1000-person-years). Attention deficit hyperactivity disorder was the most common comorbidity (24/60, 40%). ‘Stormy’ onset did not predict a worse prognosis. Global developmental delay at epilepsy onset was associated with drug resistance (*P* = 0.004, *Q* = 0.064) and with intellectual disability (*P* = 0.003, *Q* = 0.048). We found pathogenic variants in 15/39 (38.5%) patients undergoing next-generation sequencing, including four genes novel for this syndrome (*KMT2E; POGZ*; *SHANK3*; *YWHAG*), with exome sequencing yielding higher diagnostic rates than gene panels. Epilepsy with myoclonic-atonic seizures is a complex syndrome with diverse genetic causes and variable seizure severity and outcomes. Our findings expand its genetic landscape and highlight the prognostic value of prompt overall neurodevelopmental assessment at clinical onset. Whole exome sequencing should be prioritized for early diagnosis and counselling.

## Teaser

Pellacani *et al*. report that epilepsy with myoclonic-atonic seizures has diverse genetic causes, variable severity and outcomes. Early motor and language delays are strong predictors of poor long-term prognosis. Older antiseizure drugs are more effective than newer ones. Whole exome sequencing is recommended for early diagnosis and targeted counselling.

## Graphical Abstract

**Graphical Abstract:**
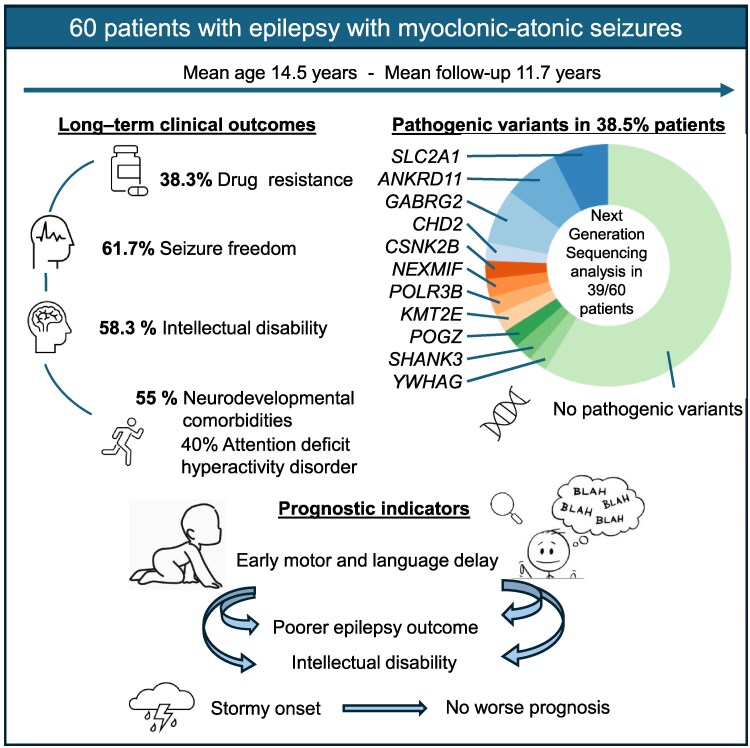

## Introduction

Epilepsy with myoclonic-atonic seizures (EMAtS), previously referred to as myoclonic-astatic epilepsy,^1^ is a generalized epilepsy syndrome of early childhood, accounting for approximately 1–2.2% of childhood-onset epilepsies.^2^

In 1970, Doose and colleagues^3^ first described a cohort of patients experiencing ‘myoclonic-astatic’ seizures, variably associated with febrile, generalized tonic-clonic, tonic and absence seizures, and episodes of non-convulsive status epilepticus (NCSE). Onset typically occurred between 1 and 7 years of age, with a peak around 3–4 years. Unlike patients with Lennox-Gastaut syndrome (LGS), these children did not show evidence of structural brain lesions and often had a positive family history of epilepsy. Reported rates of drug resistance and intellectual disability were 42 and 74%.

Subsequent studies revealed that one-third of children with this syndrome experience a ‘stormy’ onset, characterized by daily or multiple per day, generalized tonic-clonic or vibratory seizures, myoclonic-atonic seizures and NCSE episodes.^4^ In recent years, multiple monogenic aetiologies have been identified as underlying causes of what is now termed EMAtS.^5,6^

Although EMAtS is currently classified as a developmental and epileptic encephalopathy (DEE),^7^ its clinical course is highly variable. Reported outcomes range from seizure freedom within a few years of onset to long-lasting drug resistance.^4^ Cognitive outcomes also vary widely, from normal functioning to severe intellectual disability.^6,8,9^ Attention deficit hyperactivity disorder (ADHD), autism spectrum disorder (ASD) and executive function deficits are frequent comorbidities.^6,9,10^ Clinical variability complicates management of EMAtS and makes it challenging to define clear therapeutic goals.^11^

We report on a cohort of EMAtS patients with extended follow-up from two collaborating centres, with the aim of identifying prognostic factors related to both clinical presentation and genetic aetiology. Based on our findings, we also reflect on the nosological boundaries of EMAtS, especially considering that while myoclonic seizures are present in all patients, they are rarely the most disabling symptom. Nevertheless, even when marginal, they are still considered as a defining feature distinguishing EMAtS from other, otherwise similar generalized epilepsies of early childhood.

## Materials and methods

### Patients

Inclusion criteria required myoclonic-atonic seizures causing drop attacks, documented by video-EEG-EMG when available, with seizure onset between 6 months and 8 years, and interictal generalized 2–6 Hz spike-wave discharges. In a minority of patients, only myoclonic seizures were initially observed, but these were accepted as part of EMAtS if the broader clinical and EEG constellation, including EEG features, subsequent seizure evolution and age at onset, was consistent with the syndrome. We adopted this pragmatic approach to avoid underdiagnosis, acknowledging that myoclonic-atonic components may not always be demonstrable at onset, especially in settings without systematic video-EEG polygraphy. This limitation derives from the variability of the post-myoclonic EMG silent period, which constantly follows a generalized myoclonic jerk but with a duration that varies in relation to the amplitude and duration of the slow wave following the pre-myoclonic-spike.^12,13^ The post-myoclonic-atonic phenomenon, therefore, becomes clinically and electrographically apparent as such only when the silent period is long enough to cause a change in posture in relation to the subject's position and background contraction of the recorded muscles.^11,14^ Additional manifestations could include febrile, generalized tonic or tonic-vibratory, absence seizures and NCSE. Exclusion criteria included epileptic spasms, epileptic negative myoclonus causing drop attacks and seizure onset before 6 months or after 8 years. Patients were enrolled consecutively and followed prospectively from 1986 to 2024 at Meyer Children's Hospital IRCCS (Florence, Italy) and the IRCCS Stella Maris Foundation (Pisa, Italy). In addition, we conducted a retrospective chart review of all paediatric patients evaluated between 1986 and 2024, initially seen elsewhere and later referred to our centres, using electronic medical records and epilepsy databases, to identify individuals meeting the same inclusion and exclusion criteria as outlined above.

### Clinical and EEG assessment

We assessed neurodevelopment using the Alberta Infant Motor Scale^15^ and the MacArthur-Bates Communicative Development Inventories.^16^ These tools were supplemented by a detailed clinical record review to retrospectively assign developmental status at the time of seizure onset, focusing on whether motor and/or language milestones were delayed relative to normative expectations. Both centres participating in the study are dedicated to child neurology and psychiatry and provide detailed descriptions of neurological, cognitive and behavioural findings in clinical files. We conducted a comprehensive evaluation of seizures (i.e. type, frequency, onset mode, response to treatment), video-EEG recordings (i.e. background activity, epileptiform abnormalities while awake and asleep, photoparoxysmal response, electroclinical seizure semiology) and neuroimaging.

We classified different seizure types based on clinical presentation and EEG characteristics, as follows: myoclonic-atonic seizures were identified as sudden, involuntary muscular contractions involving the upper limbs or entire body, followed by a brief loss of muscle tone resulting in head drops or falls, if the patient is in any antigravity posture. The EEG correlate was a generalized spike-and-wave complex, with the spike phase time-locked to the myoclonic jerk and the slow wave phase corresponding to the atonic drop, as confirmed by EMG. Myoclonic seizures were defined as sudden, jerky, involuntary movements affecting the whole body or isolated segments, not resulting in a fall, with a time-locked EEG correlate consisting of generalized fast spike or spike-and-wave complexes.

Generalized tonic-vibratory seizures consisted of an initial rapid build-up of fast myoclonic or clonic jerks that remained stable at 5–6 Hz for several seconds before either abruptly ceasing or slowing to 1–2 Hz towards the end. This ictal pattern represents an attenuated form of classical generalized tonic-clonic seizures, in which an intense interferential EMG contraction marks the tonic phase, followed by a sustained clonic phase that concludes the episode. Absence seizures were characterized by sudden, brief lapses in awareness, with EEG showing bilateral, synchronous 1.5–2.5 Hz generalized slow spike-and-wave activity. Non-convulsive status epilepticus was defined by fluctuating unresponsiveness, intermittent myoclonic jerks and staring, with fragmentary recovery of responsiveness and motor behaviour waxing and waning over hours, accompanied by slowing of background EEG rhythms, intermingled with high-amplitude slow waves and spike-and-wave complexes.

We defined a ‘stormy’ onset as the occurrence, either at onset or within a few months after it, of clusters of frequent or repetitive generalized seizures, along with a combination of myoclonic-atonic and NCSE requiring urgent hospitalization.

All patients were treated with antiseizure medications (ASM), selected based on individual clinical and EEG profiles. Pharmacological treatments for neurodevelopmental comorbidities were also prescribed when necessary. Patients with GLUT-1 deficiency received ketogenic diet therapy. We considered patients to be seizure-free if they had not experienced any type of seizures for at least 1 year, regardless of ongoing treatment, and drug-resistant if they did not respond to at least two appropriate ASM at adequate dosages.^17^ We calculated the mortality rate as number of observed events (death) divided by the person time at risk.

### Assessment of neuropsychological and neurodevelopmental disorders

Given the long enrolment period and data originating from two centres, neuropsychological assessments varied. Most patients were prospectively and serially tested using tools available at the time of testing, including: Bayley Scales of Infant and Toddler Development,^18-20^ Columbia Mental Maturity Scale,^21^ Developmental Profile 3,^22^ Griffith Scales of Mental Development,^23^ Leiter International Performance Scale,^24-26^ Uzgiris Hunt Scale,^27^ Vineland Adaptive Behaviour Scale^28-30^ and Wechsler Intelligence Scale for Children.^31-34^ For patients who could not be formally assessed, due to young age, behavioural issues or seizure burden, we applied structured adaptive criteria, based on detailed clinical documentation, consistent with standard practice in developmental neurology.^35^ All patients underwent systematic behavioural and neurodevelopmental assessment, primarily using the Child Behaviour Checklist.^36^ In cases of suspected ADHD, or other neuropsychiatric comorbidities, additional evaluations were performed by child psychiatrists using DSM-5 criteria, with adaptations as appropriate for patients with co-occurring intellectual disability.

### Genetic analysis

After obtaining informed consent, we conducted genetic tests according to the techniques available at the time of active follow-up. We performed comparative genomic hybridization (CGH)-array analysis in 16 patients, and next-generation sequencing (NGS), including epilepsy gene panels and whole exome sequencing (WES), in 39 patients. We classified the variants according to the American College of Medical Genetics and Genomics (ACMG) criteria.

### Statistical analysis

#### Analysis of outcome predictors

We analysed as primary outcomes drug resistance, intellectual disability and other neurodevelopmental comorbidities.

We first conducted bivariate analyses using appropriate parametric tests (χ² test for categorical variables; *t*-tests for continuous variables), to explore associations between outcomes and candidate predictors (gender, family history of febrile seizures, epilepsy or both, early language and motor development, occurrence of febrile seizures, age at onset of non-febrile seizures, occurrence of a ‘stormy onset’, seizure frequency at onset—categorized into daily, weekly, monthly or annual frequency—occurrence of different seizure types and genetic aetiology).

We included in the multivariable logistic regression models the variables that demonstrated significant associations (*P* < 0.005) in single-predictor models (each adjusted for age at seizure onset and gender).

We performed a Log-rank test to examine the association between demographic and clinical variables and the prevalence of seizure freedom. To identify predictors of seizure freedom, we fitted Cox proportional hazards regression models with a single predictor of interest, adjusted for age at seizure onset and gender. For intellectual disability and neurodevelopmental comorbidities, we applied population-averaged logistic models via generalized estimating equations (GEE). This method accounts for within-subject correlation across multiple time points—in our case at 1, 2, 5, 10 and 20 years—while estimating the average effect of predictors on a binary outcome. Unlike subject-specific models such as generalized linear mixed models (GLMM), GEE focuses on population-level inference rather than individual trajectories. We used robust estimators to provide valid standard errors.

Because early delay in language and motor skills was highly correlated, we did not include them simultaneously; instead, we (i) fitted separate models for each domain and (ii) used a composite Early Developmental Delay Index (EDDI) categorizing patients as no delay, single-domain delay or dual-domain delay to capture their combined prognostic contribution while mitigating collinearity.

We applied the Benjamini–Yekutieli procedure to control the false discovery rate (FDR) under general dependence conditions, including both positive and negative correlations among test statistics. We calculated corresponding *q*-values and reported these as FDR-adjusted *P*-values.

When appropriate, we calculated confidence intervals (CIs) using likelihood. We set the level of two-sided significance at 5% (*Q* < 0.05 for adjusted analyses). We expressed results for quantitative variables as mean (±SD, range).

We performed all statistical analyses using the STATA/SE18.0 platform (StataCorp. 2023. Stata Statistical Software: Release 18. College Station, TX: StataCorp LLC).

## Results

### Patients

We included 60 patients (26.7% female) with a male to female ratio of 2.75:1. At the time of the study, the mean age was 14.5 years (±9.1, range 3.2–41), with a mean follow-up of 11.7 years (±9.4 range 0.4–40). Demographic, clinical and EEG data are summarized in Table 1.

**Table 1:** Cohort description

Demographic and clinical data	
Total	60 patients
Gender	16 females (26.7%)
Family history of epilepsy	32 patients (53.4%)
Neurodevelopment before seizure onset	Language delay only: 5 patients (8.3%)
	Motor delay only: 2 patients (3.3%)
	Both language and motor delay: 19 patients (31.7%)
	No early developmental delay: 34 patients (56.7%)
Febrile seizures	15 patients (25%)
Age at onset of febrile seizures	Mean 2.0 years (±1.1, range 0.6–3.4)
Age at other seizure onset	Any non-febrile seizure: mean 2.7 years (±1.2, range 0.8–7.2)
	MA: mean 3.1 years (±1.4, range 0.8–7.2)
	T-vib: mean 3.4 years (±1.8, range 1.0–9.1)
	M: mean 3.5 years (±2.6, range 0.8–15.3)
	ABS: mean 4.0 years (±2.8, range 0.8–12.1)
‘Stormy’ onset	26 patients (43.3%)
ICU hospitalization	1 patient (1.6%)
Seizure type prevalence	MA: 55 (91.7%)
	T-vib: 44 (73.4%)
	ABS: 30 (50%)
	M: 30 (50%)
	NCSE: 13 (21.7%)
Age at last follow-up	Mean 14.5 years (±9.1, range 3.2–41)
Remission for at least 1 year	37 patients (61.7%)
Age at seizure freedom	Mean 7.8 years (±5.17, range 2.8–28)
Duration of active epilepsy in patients in remission for at least 1 year	Mean 5.1 years (±6, range 1.2–27)
Drug-resistant patients at last follow-up	23 patients (38.4%)
Age of patients with active epilepsy at last follow-up	Mean 14.6 years (±11.1, range 3.2–41)
Follow-up duration	Mean 11.7 years (±9.4, range 0.4–40)
Mortality	1 patient (1.7%)
Mortality rate	1.80 (95% CI 0.2–12.7) per 1000-person-years
**Neuropsychological findings at last follow-up**	
Normal	25 patients (41.7%)^a^
Intellectual disability	35 patients (58.3%)^b^
**Neurodevelopmental comorbidities**	
ADHD	24 patients (40%)
Oppositional defiant disorder	8 patients (13.3%)
ASD	5 patients (8.3%)
Conduct disorder	4 patients (6.7%)
Mood disorder	4 patients (6.7%)
Anxiety disorder	3 patients (5%)

ABS, absence seizures; ADHD, attention deficit hyperactivity disorder; ASD, autism spectrum disorder; ICU, intensive care unit; M, myoclonic seizures; MA, myoclonic-atonic seizures; NCSE, non-convulsive status epilepticus; T-vib, tonic-vibratory seizures.

^a^Wechsler scale (*n* = 21), classical adaptive behavioural criteria (*n* = 4).

^b^Wechsler scale (*n* = 30), classical adaptive behavioural criteria (*n* = 5).

### Clinical and EEG findings

Thirty-two patients (53.4%) had a family history of epilepsy, febrile seizures or both, in a first-degree relative. Pregnancy and delivery were uneventful for all patients. Of the 60 patients, 5 (8.3%) had early language delay only, 2 (3.3%) had early motor delay only and 19 (31.7%) had both. In the remaining 34 (56.7%), there were no signs of early neurodevelopmental delay. Febrile seizures occurred in 15/60 patients (25%) at a median age of 2 years (±1.1, range 0.6–3.4). The mean age at onset of non-febrile seizures was 2.7 years (±1.2, range 0.8–7.2) and in 55/60 patients, seizure onset occurred after the first year of life. The mean interval between the onset of seizures and the diagnosis of EMAtS was 0.8 years (±0.8, range 0.1–2.8). We observed a ‘stormy’ onset in 26/60 patients (43.3%). During follow-up, we recorded on video-EEG clear-cut myoclonic-atonic seizures in 55/60 patients (91.7%); 25 of them also had myoclonic seizures without a detectable post-myoclonic-atonic phase. In five remaining patients, in the myoclonic seizures we recorded, a post-myoclonic EMG silent period could not be demonstrated.

**Figure 1:**
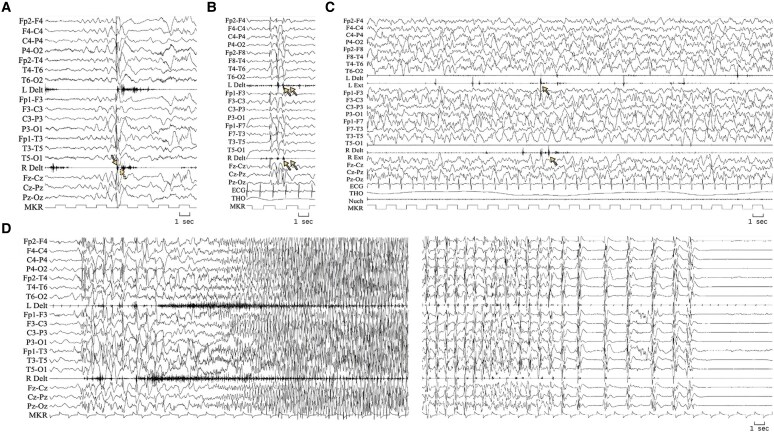
**Examples of ictal polygraphic EEG recordings.** Recording parameters: high-pass filter: 1.600 Hz; low-pass filter: 30 Hz; gain: 300 μV/cm, notch filter: 50 Hz. (**A**) Myoclonic-atonic seizure in a 27-month-old boy (Patient 41). A sudden involuntary contraction of the upper limbs is followed by a brief loss of muscle tone, resulting in arm and head brisk flexion. The EEG shows a generalized spike-and-wave complex, with the spike being time-locked to the myoclonic jerk (outlined arrow), and the slow wave corresponding to the atonic drop (dotted arrow), as confirmed in the EMG. (**B**) Myoclonic seizure in a 5-year-9-month-old boy (Patient 17). Sudden, involuntary upper limbs jerks (arrows) with time-locked generalized fast spike-and-wave complexes. (**C**) Non-convulsive status epilepticus in 3-year-old girl (Patient 58). The child manifested staring, fluctuating unresponsiveness, erratic myoclonic jerks. The EEG shows high-amplitude diffuse slow spike-and-wave activity with irregular morphology. Myoclonic potentials (arrows) are recorded from wrist extensor muscles. (**D**) Tonic-vibratory seizure in a 3-year-old boy (Patient 24). Left panel: onset of a vibratory tonic seizure with bilateral and synchronous vibratory muscle contraction accompanied by abrupt and diffuse low voltage fast activity with increasing amplitude on the EEG up to 5 Hz frequency. Right panel: end of the same seizure, showing bilateral myoclonic jerks accompanied by diffuse spike-and-wave activity with decreasing frequency. The time interval between the left panel and the right panel is 24 s. The total duration of the episode is 58 s. ECG, electrocardiogram; L Delt, left deltoid muscle; L Ext, left wrist extensor muscle; MKR, time marker (1 s); Nuch, nuchal muscle; R Delt, right deltoid muscle; R Ext, right wrist extensor muscle; THO, thoracic respiratory effort.

Forty-four patients (73.4%) had generalized tonic-vibratory seizures. Absence seizures were observed in 30/60 patients (50%) and occurred as episodes of NCSE in 13/60 (21.7%). In 33/60 patients (55%) myoclonic-atonic seizures were the initial seizure type, alone or combined with other seizure types (Fig. 1).

In all patients, interictal EEG showed 2–6 Hz generalized spike-and-wave or polyspike-and-wave discharges and in 10/60 (16.7%), a photoparoxysmal response.

**Figure 2:**
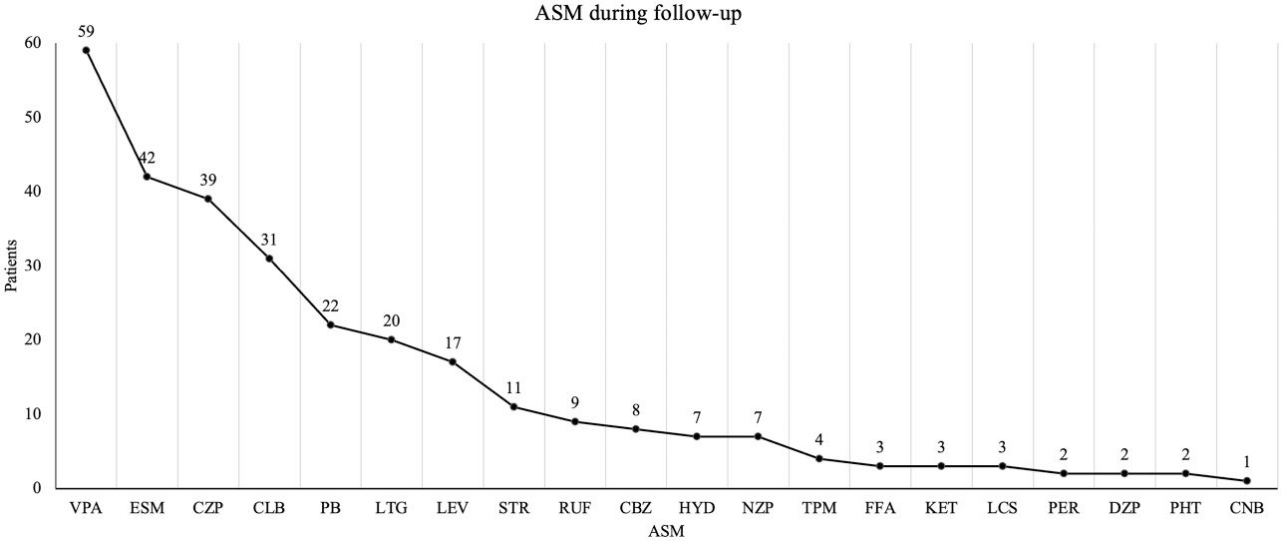
**Antiseizure medication treatment.** Number of patients treated with each antiseizure medication (ASM) during the follow-up. ASM, antiseizure medication; CBZ, carbamazepine; CLB, clobazam; CNB, cenobamate; CZP, clonazepam; DZP, diazepam; ESM, ethosuximide; FFA, fenfluramine; HYD, hydrocortisone; KD, ketogenic diet; LCS, lacosamide; LEV, levetiracetam; LTG, lamotrigine; NZP, nitrazepam; PB, phenobarbital; PER, perampanel; PHT, phenytoin; RUF, rufinamide; STIR, stiripentol; TPM, topiramate; VPA, valproic acid.

All patients received ASM, with valproate being the most used, as it was prescribed as the initial treatment in 44/60 (73.3%) and administered at some point during follow-up in 59/60 (98.3%). Among the 26 patients with ‘stormy’ onset, the most effective ASM combinations used to control closely recurrent seizures included valproate in 20/26 patients (76.9%), benzodiazepines in 18/26 (69.2%), ethosuximide in 14/26 (53.8%) and phenobarbital in 9/26 (34.6%). On average, each patient was exposed to 4.87 ASM (SD ± 2.6, range 1–12). Seizure worsening was reported in three patients (5%) with carbamazepine and in three (5%) with levetiracetam. The number of patients treated with each ASM is illustrated in Fig. 2.

Thirty-seven patients (61.7%) achieved seizure freedom at an average age of 7.8 years (SD ± 5.2, range 2.8–28), after a mean epilepsy duration of 5.1 years (SD ± 6, range 1.2–27). Five patients (8.4%) became seizure-free on monotherapy, while 32 (53.4%) required polytherapy, including three patients with GLUT-1 deficiency who also received ketogenic diet. Fourteen patients (23.4%) discontinued ASMs at an average age of 9.8 years (SD ± 3.4, range 5–15.6), with five experiencing seizure recurrence. At last follow-up, 23/60 patients (38.3%) were drug-resistant (median age 14.6 years ± 11.1, range 3.2–41). One patient (# 53) with a severe, unremitting form and no identified genetic aetiology, died in adulthood due to respiratory complications. The mortality rate in our study was 1.80 (95% CI 0.25–12.7) per 1000-person-years.

**Figure 3:**
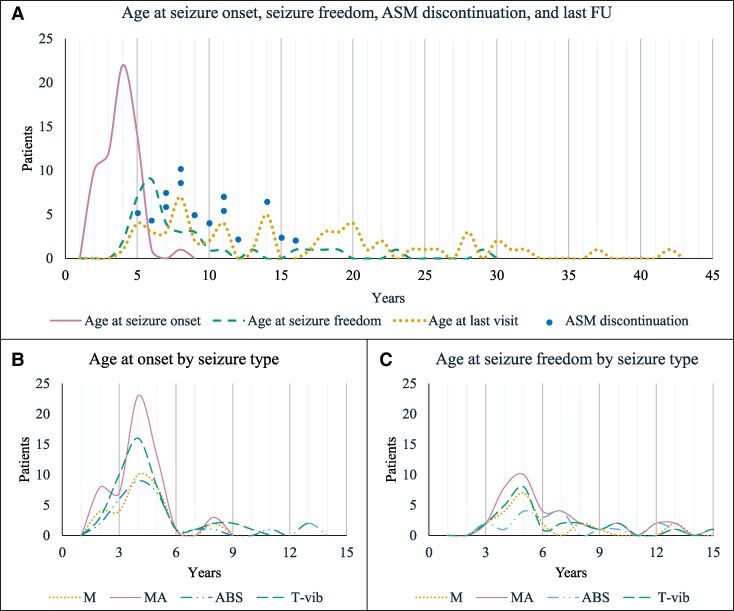
**Patients’ seizure history.** (**A**) Age at seizure onset, seizure freedom, ASM discontinuation and last FU. (**B**) Age at onset by seizure type. (**C**) Age at seizure freedom by seizure type. ABS, absence seizures; ASM, antiseizure medication; MA, myoclonic-atonic seizures; T-vib, tonic-vibratory seizures.

The distribution of patients’ ages at seizure onset and remission, along with the duration of active epilepsy and follow-up, is depicted in Fig. 3. After being diagnosed with ADHD, 5/60 patients (8.3%) were treated with methylphenidate, which was effective in four. Neuroimaging was normal in all but one patient (# 54), whose brain MRI revealed diffuse T2/FLAIR white matter hyperintensity.

### Intellectual disability and other neurodevelopmental comorbidities

At the last follow-up, 35/60 patients (58.3%) had intellectual disability. Of these, 30 had been assessed using standardized intelligence scales, revealing varying degrees of disability including mild (*n* = 19), moderate (*n* = 8) and severe (*n* = 3). Intellectual disability was present in 16/19 (84.2%) patients with both early motor and language delay versus 11/34 (32.3%) with no evidence of early delay (χ² 14.21; *P* = 0.001).

Thirty-three of 60 patients (55%) were diagnosed with one or more neurodevelopmental disorders, including ADHD in 24 (40%), oppositional defiant disorder in 8 (13.3%), ASD in 5/60 (8.3%) and conduct disorder in 4/60 (6.7%). Additionally, 4/60 patients (6.7%) were diagnosed with a mood disorder and 3/60 (5%) with an anxiety disorder. This information is summarized in Table 1.

### Genetic findings

We performed NGS in 39/60 patients (65%). Of these, 32 underwent epilepsy gene panels, which identified pathogenic variants in 8 (25%). Of 20 patients who had WES (including 13 who were mutation negative on the gene panel), 7 (35%) had a causative variant identified. While this figure suggests a higher yield than panel testing, the sequential testing strategy limits direct comparison. In total, 15/39 patients (38.5%) who underwent NGS had a pathogenic variant. CGH-array analysis, performed in 16 patients, yielded negative results.

**Figure 4:**
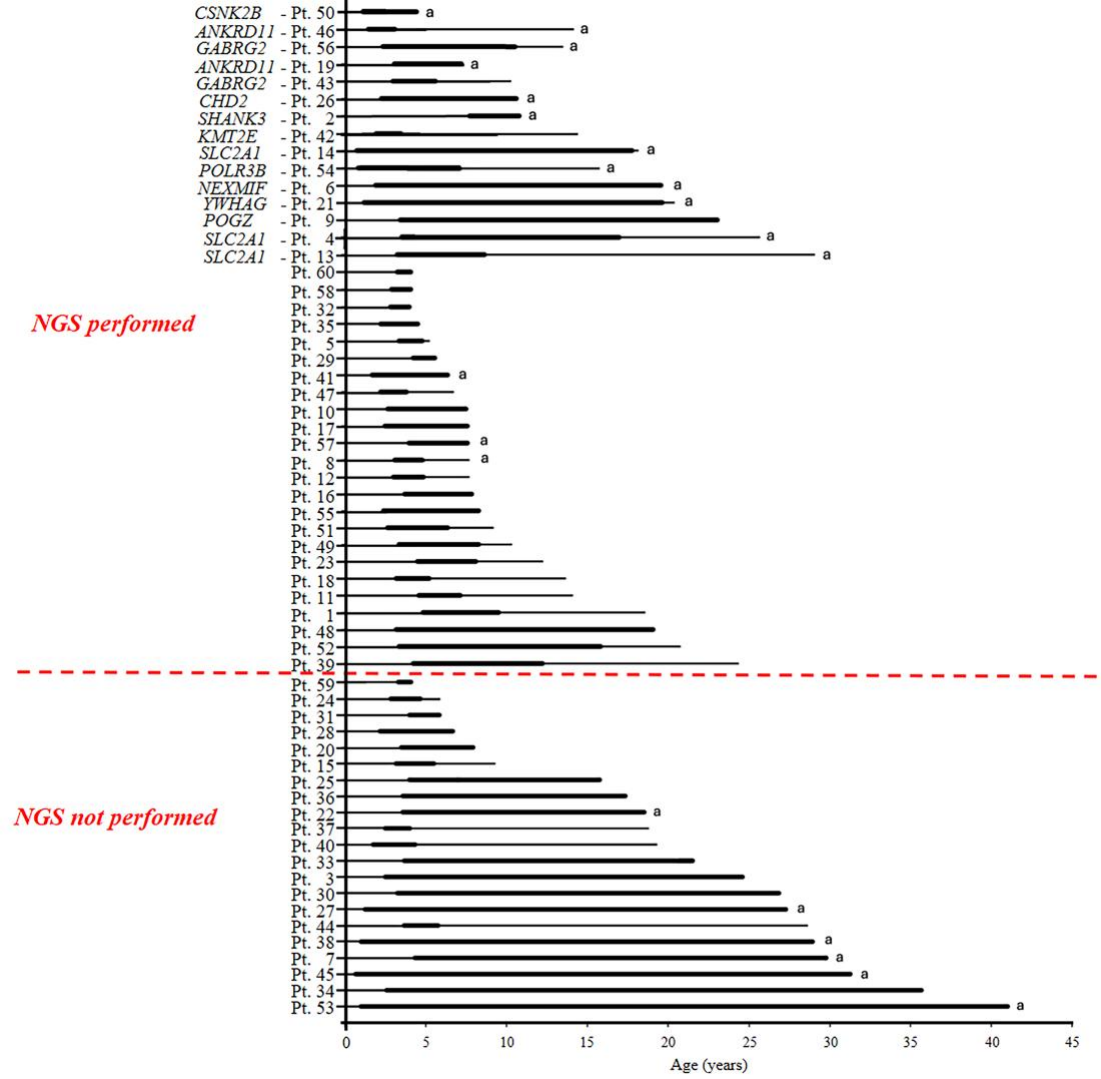
**Comparison of patients’ epilepsy course and genetic aetiology.** Each thick line represents an individual patient’s epilepsy course from seizure onset to seizure freedom (if achieved). Thin lines indicate the duration from seizure onset to the most recent follow-up for patients who did not achieve seizure freedom. ^a^Patients with motor delay before seizure onset. NGS, next-generation sequencing; Pt., patient.

We identified pathogenic variants in 11 different genes, each accounting for a single patient, including four not previously associated with EMAtS. Genetic findings are reported in Supplementary Table 1. We summarized the phenotypes of patients with the identified genetic aetiology in Supplementary Table 2. Figure 4 illustrates each patient’s seizure history and identified genetic aetiologies.

### Variables correlated with seizure and neurodevelopmental outcomes

Bivariate analyses (Supplementary Table 3) showed that early global developmental delay (both language and motor) was associated with subsequent intellectual disability (*P*  *<* 0.001). Genetic aetiology (*P*  *=* 0.006) along with myoclonic-atonic seizure at onset (*P*  *=* 0.012), were significantly correlated with intellectual disability. In contrast, tonic-vibratory seizures at onset were associated with lower prevalence of intellectual disability (*P*  *=* 0.008). We found no statistical association between ‘stormy’ onset and either seizure (*P*  *=* 0.580) or cognitive (*P*  *=* 0.536) outcomes. We found no statistical correlation between the studied variables and other neurodevelopmental comorbidities.

In single-predictor logistic regression (Table 2), we found an association between early global delay (both language and motor) with drug resistance (OR = 17.911, 95% CI: 2.500–128.303, *P*  *=* 0.004, *Q*  *=* 0.064). Global developmental delay was associated with intellectual disability (OR = 17.644, 95% CI: 2.727–114.161, *P*  *=* 0.003*, Q*  *=* 0.048), while genetic aetiology (OR = 11.004, 95% CI: 1.633–74.119, *P*  *=* 0.014*, Q*  *=* 0.211) and myoclonic-atonic seizures at onset (OR = 4.502, 95% CI: 1.497–13.539, *P*  *=* 0.007*, Q*  *=* 0.109) showed unadjusted associations but did not survive FDR correction. In multivariable models using the EDDI, global developmental delay remained associated with intellectual disability (OR = 15.068, 95% CI: 2.133–106.424, *P* = 0.007, *Q* = 0.109) after adjustment for age at onset and sex (Table 3).

**Table 2:** Single-predictor logistic regression (each model adjusted for age at seizure onset and gender): associations with drug resistance, cognitive impairment and neurodevelopmental outcomes

Variables	Drug resistance			Intellectual disability			Other developmental comorbidities		
	OR (95% CI)	*P*	*Q*	OR (95% CI)	*P*	*Q*	OR (95% CI)	*P*	*Q*
Family history of epilepsy, febrile seizure or both	0.988 (0.326–2.991)	0.982	1.000	2.393 (0.786–7.290)	0.125	1.000	0.437 (0.141–1.351)	0.151	1.000
Early language delay	8.719 (1.860–40.866)	0.006*	0.069	14.858 (2.872–76.857)	0.001*	0.013*	1.876(0.552–6.376)	0.314	1.000
Early motor delay	10.021 (2.054–48.877)	0.004*	0.047*	7.585 (1.804–31.888)	0.006*	0.069	3.086 (0.844–11–285)	0.088	1.000
EDDI									
Delay in only one domain	4.546 (0.667–30.975)	0.122	1.000	6.340 (0.989–40.632)	0.051	0.648	2.622 (0.477–14.407)	0.267	1.000
Global delay	17.911 (2.500–128.303)	0.004*	0.064	17.644 (2.727–114.161)	0.003*	0.048*	2.720 (0.673–10.978)	0.160	1.000
Febrile seizures	2.309 (0.666–8.001)	0.187	1.000	0.942 (0.285–3.112)	0.922	1.000	2.237 (0.645–7.759)	0.205	1.000
Stormy onset	1.539 (0.503–4.702)	0.450	1.000	0.466 (0.159–1.365)	0.164	1.000	1.214 (0.411–3.582)	0.726	1.000
Myoclonic-atonic seizure at onset	1.211 (0.408–3.590)	0.730	1.000	4.502 (1.497–13.539)	0.007*	0.109	1.855 (0.633–5.438)	0.260	1.000
Tonic-vibratory seizure at onset	0.895 (0.303–2.645)	0.841	1.000	0.286 (0.096–0.849)	0.024*	0.343	0.476 (0.163–1.387)	0.174	1.000
Absence seizure at onset	2.173 (0.643–7.346)	0.212	1.000	1.022 (0.312–3.351)	0.970	1.000	0.433 (0.117–1.607)	0.211	1.000
Myoclonic seizure at onset	0.546 (0.146–2.042)	0.369	1.000	0.643 (0.195–2.125)	0.470	1.000	0.564 (0.160–1.982)	0.371	1.000
Genetic aetiology	2.437 (0.480–12.366)	0.282	1.000	11.004 (1.633–74.119)	0.014*	0.211	6.270 (0.952–41.280)	0.056	0.707

EDDI, Early Developmental Delay Index (none = reference category).

^*^ *P* < 0.050; *FDR-adjusted *Q* < 0.050.

**Table 3:** Multivariable logistic regression analysis of predictors of intellectual disability (adjusted for age at seizure onset and gender)

				Gender			Age at seizure onset		
Variables	OR (95% CI)	*P*	*Q*	OR (95% CI)	*P*	*Q*	OR (95% CI)	*P*	*Q*
EDDI									
Delay in only one domain	6.535 (0.906–47.109)	0.063	0.791	0.548 (0.126–2.379)	0.422	1.000	1.282 (0.624–2.632)	0.499	1.000
Global delay	15.068 (2.133–106.424)	0.007*	0.109						
Myoclonic-atonic seizures at onset	3.735 (1.088–12.815)	0.036*	0.475						

EDDI, Early Developmental Delay Index (none = reference category).

^*^ *P* < 0.050; *FDR-adjusted *Q* < 0.050.

Compared with non-seizure-free patients, those who became seizure-free had lower rates of global developmental delay (8 versus 16.60, *P* = 0.013) and of identifiable genetic aetiology (11 versus 15.08, *P* = 0.013) (Supplementary Table 4). In univariate Cox proportional-hazard models (Table 4), we confirmed a decreased likelihood of achieving seizure freedom in patients with global developmental delay (HR = 0.090, 95% CI: 0.024–0.344, *P*  *<* 0.001*, Q*  *<* 0.001) and in patients with an identified genetic aetiology (HR = 0.290, 95% CI: 0.102–0.821, *P*  *=* 0.020*, Q*  *=* 0.292). No statistically significant associations were identified in the multivariable Cox model.

**Table 4:** Cox proportional hazards models for time to seizure freedom (single-predictor models adjusted for age at seizure onset and gender)

				Gender			Age at seizure onset		
Variables	Hazard ratio (95% CI)	*P*	*Q*	Hazard ratio (95% CI)	*P*	*Q*	Hazard ratio (95% CI)	*P*	*Q*
EDDI									
Delay in only one domain	0.446 (0.145–1.369)	0.158	1.000	2.504 (1.051–5.969)	0.038	0.498	0.570 (0.348–0.931)	0.025	0.353
Global delay	0.090 (0.024–0.344)	<0.001*	<0.001*						
Genetic aetiology	0.290 (0.102–0.821)	0.020*	0.292	2.018 (0.771–5.278)	0.152	1.000	0.880 (0.585–1.326)	0.543	1.000

EDDI, Early Developmental Delay Index (none = reference category).

^*^ *P* < 0.050; *FDR-adjusted *Q* < 0.050.

Using the composite EDDI index, GEE modelling (Supplementary Table 5) showed that global developmental delay (OR = 49.711, 95% CI: 10.424–237.069, *P*  *<* 0.001*, Q*  *<* 0.001) and genetic aetiology (OR = 11.913, 95% CI: 2.942–48.250, *P*  *=* 0.001, *Q*  *=* 0.017) were associated with higher odds of intellectual disability over time. Drug resistance (OR = 5.022, 95% CI: 1.593–15.833, *P*  *=* 0.006*, Q*  *=* 0.095) and myoclonic-atonic seizure at onset (OR = 10.886, 95% CI: 1.855–63.878, *P*  *=* 0.008*, Q*  *=* 0.124) showed non-significant trends after FDR adjustment. In multivariable GEE models (Supplementary Table 6), only global developmental delay (OR = 54.438, 95% CI: 10.296–287.817, *P*  *<* 0.001*, Q*  *<* 0.001) predicted intellectual disability over time.

## Discussion

The epilepsy syndrome, defined as EMAtS, has causal heterogeneity and includes a broad repertoire of clinical manifestations, with different types of generalized seizures and cognitive and behavioural comorbidities.^4,6,11^ From its earliest description^3^ to the most recent studies,^2^ the prognosis of EMAtS varied widely in terms of seizure outcome, cognitive function and neurodevelopmental comorbidities. Our study confirms that long-term outcomes are variable, expands the understanding of genetic causes and identifies early global neurodevelopmental delay and monogenic aetiology as key negative overall prognostic factors.

In over 90% (55/60) of patients in our cohort, seizure onset occurred after the first year of life, aligning with the ILAE definition,^7^ but more closely with the criteria proposed by Oguni.^9^ Febrile seizures preceded the onset of typical seizures in 25% of patients. Myoclonic-atonic seizures, the hallmark of the syndrome, were the initial seizure type in 55% of patients, and occurred at an average age of 3.1 years, while other seizure types typically emerged between 3.4 and 4 years.

A ‘stormy’ onset, characterized by a flurry of cluster seizures requiring urgent hospitalization, has been associated with poor prognosis by some authors,^9,37^ but evidence remains inconsistent. In our cohort, 26/60 patients (43.3%) had a ‘stormy’ onset, yet this was not associated with longer epilepsy duration (*P*  *=* 0.580) or higher rate of intellectual disability (*P*  *=* 0.536). Unlike prior reports describing ‘stormy’ phases occurring as late as 60 months after epilepsy onset,^37^ in our series it occurred at the very onset or within the initial months, hinting at severe epileptic encephalopathy, an early concern not confirmed by long-term outcome.

Our experience in this cohort indicates that the most effective drugs used during both ‘stormy’ onset and longer follow-up were valproate, ethosuximide, benzodiazepines and phenobarbital, all of which already available around the time of EMAtS original description while newly introduced ASMs have not proved effective enough to change this treatment approach. The ketogenic diet was effective in patients with GLUT-1 deficiency, if patients were able to adhere to the regimen. While the ketogenic diet has demonstrated benefit in EMAtS beyond GLUT1-deficiency, it was used in our cohort only for patients with confirmed GLUT1-deficiency syndrome. This approach reflects treatment practices over the study period, during which ketogenic diet was primarily considered in the context of a defined metabolic indication or after multiple medication failures. It also reflects a resistance to change in the eating habits of Italian families, who only in recent years, thanks to web-based boundless communication between family associations, no longer consider the ketogenic diet as an exceedingly uncomfortable option. Our findings, therefore, do not speak to the broader efficacy of ketogenic diet in EMAtS, which has been supported in other studies.^38^

Over 60% of patients achieved long-term seizure freedom. In contrast, 38.3% were drug-resistant, underscoring the syndrome’s variable trajectory. Early global neurodevelopmental delay was associated with drug resistance and intellectual disability. Our approach to evaluating early neurodevelopmental delay was grounded in identifying motor and/or language delays prior to seizure onset, using standardized assessments when available, and supplemented by detailed paediatric neurology records. We prospectively separated motor and language delays to capture domain-specific prognostic signals and, to address collinearity, also analysed a composite index (EDDI). This approach supports the view that dual-domain delay confers higher risk than single-domain delay.

While intellectual disability is the most frequently discussed outcome, our study also highlights a high prevalence (55%) of additional neurodevelopmental disorders, including ADHD, ASD, mood and anxiety disorders. These comorbidities impact long-term quality of life highlighting the need for comprehensive developmental and behavioural screening at diagnosis. The observed ADHD prevalence (40%) underscores the need for formal behavioural screening in EMAtS, especially since attentional and executive difficulties can be under-recognized in children with intellectual disability, especially when all attention is directed to seizure control. Diagnoses were made based on structured behavioural assessments and clinical judgement, considering developmental level and adaptive functioning. ADHD frequently co-occurred with mild to moderate intellectual disability, highlighting its additive impact on daily functioning and quality of life. In five patients with ADHD, we used methylphenidate, which was effective and well tolerated in four. Since neurodevelopmental comorbidities continue to impact EMAtS patients’ quality of life even after seizure remission it is crucial to consider their adequate management beyond seizure control.

We identified a genetic aetiology in 15/39 (38.5%) patients studied with NGS. WES yielded a positivity rate of 35%, which is higher than the gene panel’s rate of 25%. Since most patients who underwent WES had previously had negative panel testing, a direct comparison between the two methods remains of limited value. Pathogenic variants were distributed across 11 different genes. As previously described in EMAtS, we identified pathogenic variants in *SLC2A1* (*n* = 3), *ANKRD11* (*n* = 2), *GABRG2* (*n* = 2), *CHD2* (*n* = 1), *CSNK2B* (*n* = 1), *NEXMIF* (*n* = 1) and *POLR3B* (*n* = 1) (Supplementary Table 1), although we found no pathogenic variants in *SLC6A1*, a gene commonly associated with EMAtS.

The *POLR3B* (12q23.3, OMIM#614366) gene abnormality, observed in Patient 54, with early developmental delay, intellectual disability, ADHD and EMAtS, is consistent with previous descriptions^39,40^ but epitomizes the nosological limits of the EMAtS definition. Our patient, as well as 1/3 described by Djordjevic *et al.*^39^ and 2/7 described by Symonds *et al.*^40^ exhibited diffuse hypomyelination, while an identical EMAtS-consistent phenotype was associated with a normal brain MRI in 2/3 patients of Djordjevic *et al.*^39^ and in 5/7 of Symonds *et al.*^40^ These observations highlight the limitations associated with narrow nosological definitions of EMAtS and the challenges posed by the identification of a syndromic phenomenology with a presumed non-lesional aetiology. For instance, a patient with normal brain MRI is accepted as having EMAtS, understood as a non-lesional form of generalized epilepsy, but is excluded if the MRI is abnormal, even when the genetic aetiology is the same.

We identified variants in four additional genes that had not previously been associated with EMAtS. *SHANK3* (22q13.33, OMIM#606230) pathogenic variants are associated with seizures in 60% of patients,^41^ with no specific epilepsy syndromes being reported. Patient 2 had global developmental delay at seizure onset, severe intellectual disability, ASD and drug-resistant EMAtS starting at age 6 years. *POGZ* (1q21.3, OMIM#614787) pathogenic variants are associated with hypotonia, dysmorphisms, microcephaly, developmental delay, ASD and epilepsy in 20% of patients.^42^ In Patient 9, the *POGZ* variant was associated with moderate intellectual disability and drug-resistant EMAtS. *YWHAG* (7q11.23, OMIM#605356) phenotypic spectrum includes mild delay to DEE, with generalized seizures (100%), intellectual disability (96%), behavioural disorders (75%), neurological signs (54%) and dysmorphisms (25%).^43^ Patient 21 exhibited typical EMAtS comorbid with severe intellectual disability. *KMT2E* (7q22, OMIM#618884) pathogenic variants are associated with macrocephaly, mild intellectual disability, ASD and, in one-third of patients, unclassified epilepsy.^44^ Patient 42 had EMAtS onset at age 2 years, became seizure-free at 10 years and had borderline IQ scores when last seen at 13 years.

The identified genetic aetiologies can be grouped based on their known biological roles and include genes involved in synaptic function and neuronal signalling (e.g. *SHANK3*, *NEXMIF*, *SYNGAP1*, *STXBP1*, *POGZ*), transcriptional and chromatin regulation (e.g. *CHD2*, *CSNK2B*, *ANKRD11*, *KMT2E*, *POGZ*, *POLR3B*), receptors (e.g. *GABRB3*, *GABRG2*), ion channels (e.g. *SCN2A, SCN1A, KCNT1*), ion transporters (e.g. *SLC2A1, SLC6A1*) and cellular differentiation and neuronal function (e.g. *YWHAG, SEMA6B*) (Supplementary Table 1). This categorization helps highlight shared pathways, such as synaptic transmission and chromatin remodelling, which may converge on common neurodevelopmental vulnerabilities contributing to EMAtS phenotypes, but also underscores the convergent phenotype of EMAtS across a diverse range of molecular mechanisms.

In patients with an identified genetic aetiology, we observed a higher rate of intellectual disability (*P*  *=* 0.014*, Q*  *=* 0.211) consistent with previous studies.^2,6^ Among mutation-negative patients, some might still harbour single gene abnormalities that escaped recognition, while others might not have a monogenic disorder.

In view of the extreme genetic heterogeneity, WES should ideally be included in the early diagnostic workflow of EMAtS, as obtaining a genetic diagnosis may provide prognostic insights and highlight rare, potentially actionable aetiologies such as *SLC2A1,*^38^  *SLC6A1*^45,46^ and *CHD2*^47^ pathogenic variants.

Historically, EMAtS has been considered a syndrome of previously normally developing children with normal brain imaging. However, recent studies and our findings highlight that some patients meeting full electroclinical criteria for EMAtS may present with early neurodevelopmental delays or neuroimaging abnormalities. For example, one patient in our series with a pathogenic *POLR3B* variant exhibited white matter signal changes yet had an EMAtS phenotype. These patients challenge the strict application of ‘normal development and imaging’ as inclusion criteria, suggesting the need for a more flexible, phenotype-driven approach, particularly in genetically defined cohorts. As the field shifts towards genetic-first and aetiology-based classification, some patients meeting full electroclinical criteria for EMAtS may also fulfil the definition of a DEE, particularly when associated with moderate/severe developmental delay or a known genetic diagnosis (e.g. *SYNGAP1, SCN2A*). The question of whether these patients should be considered within or adjacent to the EMAtS spectrum remains open. Our data support the view that EMAtS-like phenotypes can occur within broader DEEs, and that the current nosological boundary may be too rigid to capture these overlaps. Our findings concur with prior work, including the Tang *et al*. (2020; *n* = 101) multicentre cohort,^6^ that developmental delays preceding epilepsy onset are associated with a higher probability of identifying a genetic aetiology in EMAtS phenotypes. Within the ILAE framework, early multi-domain delays and other ‘alerts’ such as neuroimaging abnormalities or severe intellectual disability, should therefore raise suspicion for a monogenic cause and prioritize comprehensive genomic testing, ideally WES.

Our inclusion of patients with myoclonic seizures without a demonstrable atonic component reflects a pragmatic approach derived from long-term clinical experience and awareness of the pathophysiology of generalized epileptic myoclonus which indicates how the clinical distinction between a myoclonic-atonic and a myoclonic phenomenon lies purely in a variation in intensity and duration of the post-myoclonic EMG silent period.^11,12^ We recognize that this departs from the strict ILAE requirement of mandatory myoclonic-atonic seizures, but believe it captures real-world cases in which atonic elements are either subtle, only manifest later or not captured by available EEG recordings.

EMAtS exhibits a phenotypic pattern independent of a specific aetiology. Variable aetiologies are also observed in other age-related syndromes appearing in the same age range and featuring different types of generalized seizures, such as LGS and LGS-like conditions. However, EMAtS, unlike LGS, tend not to appear in association with macroscopic structural brain lesions and much more often has a favourable outcome. This suggests that, for EMAtS to manifest, brain anatomy and function must be at least relatively spared. For both syndromes, only a fragment of the typical phenotype can be observed in some patients. When incomplete forms of LGS are manifested, they are designated as symptomatic generalized epilepsies or, more recently, DEEs.^48^ Likewise, it is not uncommon to observe children who exhibit only fragments of the full blown EMAtS phenotype. In line with the current ILAE classification, myoclonic-atonic seizures remain mandatory for an EMAtS diagnosis; patients who never develop this seizure type should not be classified within the syndrome, even if other features overlap. Children who present with other combinations of difficult-to-treat generalized seizures but no myoclonic-atonic seizures are better considered within the spectrum of DEE, or previously in the symptomatic generalized epilepsies. However, no studies have explored to what extent, if only myoclonic-atonic seizures are missing, aetiologies and outcomes are indicative of clinical entities that lie outside the spectrum comprising EMAtS. This knowledge gap suggests that the current definition of EMAtS—centred around the myoclonic-atonic phenomenon—may be overly restrictive, possibly an artefact of its historical framing based on myoclonic seizures having been identified as the defining feature.^11^ A similar process occurred with Dravet syndrome, which was originally identified as severe myoclonic epilepsy in infancy,^49^ and then renamed to its current denomination after realizing that myoclonus is not present in all individuals and, even when present, is not the most prominent clinical manifestation of the syndrome. However, unlike Dravet syndrome, now recognized as associated only with pathogenic variants in the *SCN1A* gene, EMAtS remains genetically heterogeneous.

We acknowledge some limitations of this study. Patients were recruited from two tertiary paediatric neurology centres, potentially leading to a recruitment bias favouring more severe, complex or drug-resistant patients. As such, caution is warranted in generalizing these findings to broader community-based populations. Not all patients underwent complete formal neuropsychological testing, and variability in assessment tools over time may have affected the consistency of cognitive outcome measurements. Retrospective application of motor and language delay criteria, though based on prospectively gathered, detailed specialist documentation, may be subject to documentation and recall bias. While this approach introduced an element of subjectivity, it allowed us to include patients who would otherwise be excluded from outcome analysis. We acknowledge that missing data and tool variability may have affected statistical power and introduced recall or documentation bias. These limitations are inherent to retrospective designs spanning decades but were mitigated by the systematic use of GEE to increase analytical robustness. Only 65% of patients underwent NGS, reflecting evolving diagnostic availability over the study period. WES was often performed after negative gene panel results, which may overestimate the comparative yield of WES. Furthermore, while we classified variants using ACMG criteria and integrated phenotypic data, we did not perform functional validation for the novel variants. Therefore, these novel associations should be interpreted as candidate gene findings, requiring further confirmation in functional or independent cohort studies.

EMAtS is a clinically and genetically heterogeneous syndrome with variable seizure outcomes and frequent neurodevelopmental comorbidities. Our findings highlight the prognostic value of early global development, support the prioritization of WES and reveal novel genetic associations. Future research should focus on refining the clinical boundaries of EMAtS, validating predictive models in independent cohorts and exploring mechanistic insights into newly implicated genes. Longitudinal studies are also needed to assess the impact of targeted behavioural interventions, particularly for ADHD and executive dysfunction, on long-term quality of life.

## Supplementary Material

fcaf507_Supplementary_Data

## Data Availability

The data that support the findings of this study are available from the corresponding author upon reasonable request.
