# Supplementary material for: Epilepsy with myoclonic-atonic seizures: genetic aetiologies, outcomes and prognostic indicators
Source: Brain Commun. 2025 Dec 29;8(1):fcaf507. doi: 10.1093/braincomms/fcaf507 (PMC12782104; doi:10.1093/braincomms/fcaf507)
Supplement: fcaf507_Supplementary_Data [file fcaf507_supplementary_data.doc]

**Supplementary Table 1. Published genetic variants associated with EMAtS, grouped by functional role, including novel findings from this series (bolded)**

| **Approved symbol** | **Gene/locus name** | ***n* of patients reported** | **Variants (*n* of times each variant is reported)** | | **Inheritance**  **(*n* of patients reported for each mode of inheritance)** | | **References**  **(*n* of patients reported)** |
| --- | --- | --- | --- | --- | --- | --- | --- |
| **Transcriptional and Chromatin regulation** | | | |  | |  | |
| *ANKRD11* | Ankyrin repeat domain–containing protein 11 | 7 | c.5145C>G; p.(Tyr1745Ter) (1)  c.5659C>T; p.(Gln1887Ter) (1)  c.6836_6837delTG; p.(Val2279GlyfsTer16) (1)  c.6982dup; p.(Arg2328fs) (1)  c.7874_7877delAGCT (1)  **c.6836_6837delTG; p.(Val2279GlyfsTer16) (1)**  **c.142G>A; p.(Gly48Arg)** **(1)** | | *De novo* (3)  NA (2)  ***De novo* (2)** | | Buijsse *et al.*^1^ (2)  Alves *et al.*^2^ (1)  Auconi *et al.*^3^ (1)  Whitney *et al.*^4^ (1)  **This series (2)** |
| *CHD2* | Chromodomain helicase DNA binding protein 2 | 8 | c.1861C>T; p.(Arg621Trp) (1)  c.4931_4932delGA; p.(Arg1644LysfsTer22) (1)  c.3067-2A>G (1)  c.4233_4236delAGAA; p.(Glu1412GlyfsTer64) (1)  c.4256del19; p.(Lys1419fsTer3) (1)  c.5120G>A; p.(Arg1707Gln) (1)  NA (1)  **c.2095C>T; p.(Arg699Trp)** **(1)** | | *De novo* (5)  NA (2)  ***De novo* (1)** | | Carvill *et al.*^5^ (2)  Trivisano *et al.*^6^ (1)  Chen *et al.*^7^ (1)  Angione *et al.*^8^ (1)  Routier *et al.*^9^ (1)  Nickels *et al.*^10^ (1)  **This series (1)** |
| *CSNK2B* | Beta subunit of casein kinase II | 3 | c.303C>A; p.(Tyr101Ter) (2)  **c.408C>A; p.(Tyr136Ter)** **(1)** | | NA (2)  ***De novo* (1)** | | Ernst *et al.*^11^ (2)  **This series (1)** |
| *FTSJ1* | Ftsj RNA methyltransferase homolog 1 | 1 | c.9+1G>T (1) | | MAT (1) | | Routier *et al.*^9^(1) |
| *HNRNPU* | Heterogeneous nuclear ribonucleoprotein U | 2 | c.878A>G; p.(Tyr293Cys) (1)  c.691C>T; p.(Arg231Ter) (1) | | *De novo* (2) | | Kim *et al.*^12^ (1)  Hinokuma *et al.*^13^ (1) |
| ***KMT2E**** | Lysine methyltransferase 2E | 1 | **c.4997C>T; p.(Ser1666Leu) (1)** | | ***De novo* (1)** | | **This series (1)** |
| *MECP2* | Methyl-CpG binding protein 2 | 2 | c.259C>A; p.(Pro87Thr) (1)  c.673C>A; p.(Pro225Thr) (1) | | *De novo* (2) | | Tang *et al.*^14^ (1)  Routier *et al.*^9^ (1) |
| ***POGZ**** | Pogo transposable element with zinc finger domain | 1 | **c.3283A>G; p.(Lys1095Glu)** **(1)** | | ***De novo* (1)** | | **This series (1)** |
| *POLR3B* | RNA polymerase subunit B | 11 | c.1072G>T; p.(Gly358Cys) (1)  c.1087G>A; p.(Glu363Lys) (1)  c.1094C>T; p.(Ala365Val) (2)  c.1124A>G; p.(Asp375Gly) (1)  c.1277T>C; p.(Leu426Ser) (2)  c.1304G>T; p.(Gly435Val) (1)  c.1373A>C; p.(Gln458Pro) (1)  c.1729A>G; p.(Asn577Asp) (1)  **c.2944A>G; p.(Asn982Asp)/c.1903G>A; p.(Val635Met) (1)** | | *De novo* (9)  NA (1)  **AR (1)** | | Djordjevic *et al.*^15^ (3)  Symonds *et al.*^16^ (7)  **This series (1)** |
| *SMARCA2* | SWI/SNF-related, matrix associated, actin-dependent regulator of chromatin, subfamily A, member 2 | 1 | c.3721C>G; p.(Gln1241Glu) (1) | | *De novo* (1) | | Tang *et al.*^17^ (1) |
| **Ion channels** | |  |  | |  | |  |
| *CACNA1H* | Calcium channel, voltage-gated T-type, alpha 1H subunit | 2 | c.3283G>A; p.(Asp1095Asn)/c.4756A>G; p.(Arg1586Gly) (1)  NA (1) | | NA (1)  CH (1) | | Routier *et al.*^9^ (1)  Nickels *et al.*^10^ (1) |
| *KCNA2* | Potassium channel, voltage-gated Shaker-related subfamily, member 2 | 3 | c.788T>C; p.(Ile263Thr) (2)  c.889C>T; p.(Arg297Trp) (1) | | *De novo* (3) | | Syrbe *et al.*^18^ (1)  Masnada *et al.*^19^ (1)  Routier *et al.*^9^ (1) |
| *KCNB1* | Potassium channel, voltage-gated, shab-related subfamily, member 1 | 1 | c.916C>T; p.(Arg306Cys) (1) | | *De novo* (1) | | Tang *et al.*^14^(1) |
| *KCNT1* | Potassium channel, subfamily T, member 1 | 1 | c.862G>A; p.(Gly288Ser) (1) | | *De novo* (1) | | Routier *et al.*^9^(1) |
| *SCN1A* | Sodium channel, voltage-gated, type 1, alpha polypeptide | 2 | c.1299insC; p.(Leu434ProfsTer16) (1)  c.3521C>G; p.(Thr1174Ser) (1) | | *De novo* (1)  MAT (1) | | Ebach *et al.*^20^ (1)  Yordanova *et al.*^21^ (1) |
| *SCN2A* | Sodium channel, voltage-gated, type II, alpha subunit | 2 | c.2790C>A; p.(His930Gln) (1)  c.4864C>T; p.(Pro1622Ser) (1) | | *De novo* (2) | | Wolff *et al.*^22^ (2) |
| *SCN8A* | Sodium channel, voltage gated, type VIII, alpha polypeptide | 1 | NA (1) | | NA (1) | | Kim *et al.*^23^ (1) |
| **Synapse-related** | |  |  | |  | |  |
| *AP2M1* | Adaptor-related protein complex 2, mu 1 subunit | 1 | c.508C>T; p.(Arg170Trp) (1) | | *De novo* (1) | | Helbig *et al.*^24^(1) |
| *IQSEC2* | Guanine nucleotide exchange factor for the ARF family of GTP-binding proteins | 1 | c.136G>T; p.(Glu46Ter) (1) | | *De novo* (1) | | Kim *et al.*^12^(1) |
| *NEXMIF* | Neurite extension and migration factor | 10 | c.964C>T; p.(Arg322Ter) (1)  c.1261_1270del; p.(Leu421ProfsTer) (1)  NA (7)  **c.1262_1271del; p.(Leu421GlnfsTer76) (1)** | | *De novo* (1)  NA (8)  ***De novo* (1)** | | Stamberg *et al.*^25^ (7)  De Lange *et al.*^26^ (2)  **This series (1)** |
| ***SHANK3**** | SH3 and multiple ankyrin repeat domains protein 3 | 1 | **c.4020_4021del; p.(Asp1340fs) (1)** | | **NA (1)** | | **This series (1)** |
| *STX1B* | Syntaxin 1B | 2 | c.676G>C; p.(Gly226Arg) (1)  c.733C>T; p.(Arg245Ter) (1) | | *De novo* (2) | | Schubert *et al.*^27^ (1)  Burghardt *et al.*^28^ (1) |
| *STXBP1* | Syntaxin binding protein 1 | 1 | c.288T>C; UTR (1) | | *De novo* (1) | | Routier *et al.*^9^ (1) |
| *SYNGAP1* | Synaptic Ras GTPase activating protein 1 | 6 | c.274_277del ; p.(Gly92AlafsTer) (1)  c.1735C>T; p.(Arg579Ter) (1)  c.1995T>A ; p.(Tyr665Ter) (1)  c.2176_2179del; p(Arg726GlufsTer) (1)  c.2562_2578del; p.(Arg854AlafsTer) (1)  c.3583-6G>A; p.(Val1195AlafsTer27) (1) | | *De novo* (5)  NA (1) | | Mignot *et al.*^29^ (3)  Berryer *et al.*^30^ (1)  Liu *et al.*^31^ (1)  Lo Barco *et al.*^32^ (1) |
| **Cellular differentiation and neuronal function** | | | |  | |  | |
| *KLC2* | Kinesin light chain 2 | 1 | c.1295G>A; p.(Gly432Glu)/c.1785+42G>T (1) | | CH (1) | | Routier *et al.*^9^(1) |
| *SEMA6B* | Semaphorin 6B | 1 | c.2138C>T; p.(Thr713Met) (1) | | *De novo* (1) | | Cordovado *et al.*^33^(1) |
| *SHROOM4* | Shroom family member 4 | 1 | c.1201C>T ; p.(His401Tyr) (1) | | NA (1) | | Routier *et al.*^9^(1) |
| *SUN1* | Sad1 and UNC84 domain-containing protein 1 | 1 | c.1779+2_1779+9dup/c.956C>G; p.(Pro319Arg) (1) | | CH (1) | | Routier *et al.*^9^ (1) |
| ***YWHAG**** | Tyrosine 3-monooxygenase/tryptophan 5-monooxygenase activation protein, gamma subunit | 1 | **c.394C>G; p.(Arg132Gly) (1)** | | ***De novo* (1)** | | **This series (1)** |
| **Receptors** | |  |  | |  | |  |
| *GABRB3* | Gamma-aminobutyric acid (GABA) A receptor, beta-3 | 7 | c.8delG; p.(Gly3AlafsTer26) (1)  c.227C>G; p.(Ser76Cys) (1)  c.331C>T; p.(Arg111Ter) (1)  c.358G>A; p.(Asp120Asn) (2)  c.425G>T; p.(Arg142Leu) (1)  c.550T>C; p.(Tyr184His) (1) | | *De novo* (4)  MAT (3) | | Møller *et al.*^34^ (5)  Epi4k consortium^35^ (1)  Yang *et al.*^36^ (1) |
| ***GABRG2*** | Gamma-aminobutyric acid (GABA) A receptor, gamma-2 subunit | 3 | c.770-1G>A (1)  **c.821A>G; p.(Tyr274Cys) (1)**  **c.1042+1G>A (1)** | | NA (1)  **NA (2)** | | Angione *et al.*^8^(1)  **This series (2)** |
| **Transporters** | | |  | |  | |  |
| *SLC6A1* |  | 39 | c.46G>T; p.(Glu16Ter) (1)  c.131G>A; p.(Arg44Gln) (1)  c.149G>T; p.(Arg50Leu) (1)  c.226A>G; p.(Lys76Glu) (1)  c.236G>T; p.(Gly79Val) (1)  c.419A>G; p.(Tyr140Cys) (2)  c.491G>A; p.(Cys164Tyr) (1)  c.518G>A; p.(Cys173Tyr) (1)  c.578G>A; p.(Trp193Ter) (1)  c.695G>T; p.(Gly232Val) (2)  c.739C>G; p.(Pro247Ala) (1)  c.815_817delTCA; p.(Ile272del) (1)  c.850-2A>G (1)  c.863C>T; p.(Ala288Val) (3)  c.871C>T; p.(Gln291Ter) (1)  c.881-883del; p.(Phe294del) (2)  c.889G>A; p.(Gly297Arg) (1)  c.912C>G (1)  c.987C>А; p.(Cys329Ter) (1)  c.1000G>C; p.(Ala334Pro) (1)  c.1024G>A; p.(Val342Met) (3)  c.1070C>T; p.(Ala357Val) (2)  c.1079-1G>A; p.(Gly360ValfsTer14) (1)  c.1084G>A; p.(Gly362Arg) (1)  c.1155C>G; p.(Phe385Leu) (2)  c.1342A>T; p.(Lys448Ter) (1)  c.1369_1370delGG; p.(Gly457HisfsTer10) (1)  c.1379T>G; p.(Leu460Arg) (1)  c.1485G>A; p.(Trp495Ter) (1)  c.1600C>T; p.(Gln534Ter) (1) | | *De novo* (31)  MAT (4)  PAT (2)  NA (2) | | Johannesen *et al.*^37^ (14)  Carvill *et al.*^38^ (7)  Mermer *et al.*^39^ (4)  Silva *et al.*^40^ (3)  Tang *et al.*^14^ (2)  Goodspeed *et al.*^41^ (2)  Palmer *et al.*^42^ (1)  Zech *et al.*^43^ (1)  Kim *et al.*^12^ (1)  Mattison *et al.*^44^ (1)  Hinokuma *et al.*^13^ (1)  Yuan *et al.*^45^ (1)  Panda *et al.*^46^ (1) |
| *SLC2A1* | Solute carrier family 2 (facilitated glucose transporter), member 1 | 10 | c.348delC; p.(Lys117SerfsTer6) (1)  c.376C>T; p.(Arg126Cys) (1)  c.940G>C; p.(Gly314Arg) (1)  c.971C>T; p.(Ser324Leu) (1)  c.997C>T; p.(Arg333Trp) (2)  c.1199G>A; p.(Arg400His) (1)  **c.997C>T; p.(Arg333Trp) (2)**  **c.1199G>A; p.(Arg400His) (1)** | | *De novo* (3)  NA (2)  MAT (2)  ***De novo* (2)**  **NA (1)** | | Mullen *et al.*^47^ (4)  Simard-Tremblay *et al.*^48^ (1)  Lebon *et al.*^49^ (1)  Kim *et al.*^12^ (1)  **This series (3)** |
| **Protein biosynthesis/degradation-related** | | | |  | |  | |
| *TPP1* | Tripeptidyl peptidase 1 | 1 | c.509-1G>A / c.622C>T; p.(Arg208Ter) (1) | | CH (1) | | Routier *et al.*^9^ (1) |
| **Copy number variants (CNV)** | | | |  | |  | |
| t(3;11)(p25;q13.1)dn |  | 1 | 3p25.3 translocation breakpoint in the intron 7 of *SLC6A1* (1) | | *De novo* (1) | | Mori *et al.*^50^ (1) |
| 16p11.2 del |  | 1 | 1.2 Mb del (including *STX1B)* (1) | | *De novo* (1) | | Vlaskamp *et al.*^51^ (1) |
| 2q24.3 del |  | 1 | 588.7 Kb del (including *TTC21B*, *SCN1A*, *SCN9A*, *SCN7A)* (1) | | *De novo* (1) | | Hinokuma *et al.*^13^ (1) |
| Xp22.31 del |  | 1 | 300 Kb del (including *PUDP* and *STS*) (1) | | *De novo* (1) | | Hinokuma *et al.*^13^ (1) |
| 16p13.3 dup |  | 1 | 407 Kb dup (including *TBC1D24*) (1) | | NA (1) | | Balestrini *et al*.^52^ (1) |

AR=autosomal recessive; CH = compound heterozygous; CNV = copy number variants; MAT = maternal inheritance; *n* = number; NA = not available; PAT = paternal inheritance; ******* not previously associated with EMAtS.

**Supplementary Table 2. Neurodevelopmental phenotype and age at onset of each seizure type of patients with identified genetic aetiology included in this study (in decreasing order of frequency).**

| **Gene variant** | **Patient** | **Age at seizure onset / Type** | **Early development** | **Neuropsychological and neurodevelopmental phenotype** |
| --- | --- | --- | --- | --- |
| *SLC2A1*  NM_006516.4: c.997C>T; p.(Arg333Trp) | Patient 4 | 3.4 years / MA  1year / ABS | Global developmental delay | Severe ID, language impairment (last tested at age 15) |
| *SLC2A1*  NM_006516.4: c.997C>T; p.(Arg333Trp) | Patient 13 | 3 years / MA | Motor developmental delay | Severe ID, school support (last tested at age 13) |
| *SLC2A1*  NM_006516.4: c.1199G>A; p.(Arg400His) | Patient 14 | 0.8 years / MA  12.1 years / ABS | Global developmental delay | Mild ID, ADHD, school support (last tested at age 16) |
| *ANKRD11*  NM_001256182.1 :c.142G>A; p.(Gly48Arg) | Patient 19 | 2.8 years / MA  2.8 years / ABS | Global developmental delay | ID, school support (last tested at age 7) |
| *ANKRD11*  NM_013275.6: c.6836_6837delTG; p.(Val2279GlyfsTer16) | Patient 46 | 1.3 years / MA | Global developmental delay | Mild ID, language impairment, ADHD (last tested at age 12) |
| *GABRG2*  NM_000816.3: c.821A>G; p.(Tyr274Cys) | Patient 43 | 2.5 years / MA | Unknown (adopted) | Moderate ID, ADHD, school support (last tested age 4) |
| *GABRG2*  NM_198903.2: c.1042+1G>A | Patient 56 | 2.2 years / MA  2.2 years / T-vib  2.2 years / NCSE | Global developmental delay | Normal cognitive function, ADHD (last tested at age 11) |
| *CHD2*  NM_001271.4: c.2095C>T; p.(Arg699Trp) | Patient 26 | 2.1 years / MA  2.1 years / ABS  8 years / T-vib | Global developmental delay | Moderate ID, language impairment, school support (last tested at age 7) |
| *CSNK2B*  NM_001320.5: c.408C>A; p.(Tyr136Ter) | Patient 50 | 0.8 years / MA  1 years / T-vib | Global developmental delay | Mild ID, language impairment (last tested at age 3) |
| *KMT2E ^*  NM_018682.3: c.4997C>T; p.(Ser1666Leu) | Patient 42 | 2 years / MA  2 years / ABS  2 years / NCSE  2 years / T-vib | Normal early development | Borderline cognitive function (last tested at age 13) |
| *NEXMIF*  NM_001008537.2: c.1262_1271del; p.(Leu421GlnfsTer76) | Patient 6 | 1.8 years / MA  1.8 years / ABS | Global developmental delay | Moderate ID (last tested at age 17) |
| *POLR3B*  NM_018082.3: c.2944A>G; p.(Asn982Asp) NM_018082.3: c.1903G>A; p.(Val635Met) | Patient 54 | 0.9 years / MA  0.9 years / M  3.3 years / T-vib | Global developmental delay | Moderate ID, ADHD, school support (last tested at age 9) |
| *POGZ* ^  NM_015100.4: c.3283A>G; p.(Lys1095Glu) | Patient 9 | 3.3 years / MA  3.3 years / ABS  3.3 years / T-vib  6.5 years / NCSE | Normal early development | Moderate ID, language impairment, ADHD, school support (last tested at age 20) |
| *SHANK3 ^*  NM_001372044.2: c.4020_4021del; p.(Asp1340fs) | Patient 2 | 6 years / MA  6 years / T-vib | Global developmental delay | Severe ID, ASD, language impairment, school support (last tested at age 4) |
| *YWHAG ^*  NM_012479.3: c.394C>G; p.(Arg132Gly) | Patient 21 | 1.8 years / MA  1.8 years / T-vib | Global developmental delay | Severe ID, language impairment, school support (last tested at age 16) |

ABS = absence seizures; ADHD = attention deficit hyperactivity disorder; ASD = autism spectrum disorder; ID = intellectual disability; M = myoclonic seizures; MA = myoclonic-atonic seizures; NCSE = non-convulsive status epilepticus; T-vib = tonic-vibratory seizures.

***^*** =not previously associated with EMAtS.

**Supplementary Table 3. Bivariate associations with outcomes (drug resistance, intellectual disability and other neurodevelopmental comorbidities) using parametric Χ^2^ test and two-sample *t-*test.**

| **Variables** | **Drug Resistance** | | | **Intellectual disability** | | | **Other neurodevelopmental comorbidities** | | |
| --- | --- | --- | --- | --- | --- | --- | --- | --- | --- |
|  | **No** | **Yes** | ***P-* value^a^** | **Normal** | **ID** | ***P-* value^a^** | **No** | **Yes** | ***P-* value^a^** |
| Gender |  |  |  |  |  |  |  |  |  |
| Male | 30 | 14 | 0.085 | 18 | 26 | 0.843 | 16 | 28 | 0.340 |
| Female | 7 | 9 |  | 7 | 9 |  | 8 | 8 |  |
| Family history of febrile seizures and/or epilepsy | | | |  |  |  |  |  |  |
| No | 16 | 10 | 0.867 | 15 | 11 | 0.196 | 13 | 13 | 0.147 |
| Yes | 19 | 13 |  | 13 | 19 |  | 22 | 10 |  |
| Early language Development |  |  |  |  |  |  |  |  |  |
| Normal | 26 | 10 | 0.039* | 23 | 13 | <0.001* | 18 | 18 | 0.052 |
| Delayed | 11 | 13 |  | 2 | 23 |  | 6 | 18 |  |
| Early motor Development |  |  |  |  |  |  |  |  |  |
| Normal | 28 | 11 | 0.028* | 24 | 20 | <0.001* | 12 | 13 | 0.793 |
| Delayed | 9 | 12 |  | 1 | 15 |  | 18 | 17 |  |
| EDDI^ |  |  |  |  |  |  |  |  |  |
| Normal | 25 | 9 | 0.076 | 23 | 11 | 0.001* | 23 | 11 | 0.241 |
| Delay in only one domain (language or motor) | 4 | 3 |  | 2 | 5 |  | 3 | 4 |  |
| Global delay (both language and motor) | 8 | 11 |  | 3 | 16 |  | 9 | 10 |  |
| Febrile seizures |  |  |  |  |  |  |  |  |  |
| No | 30 | 15 | 0.168 | 18 | 27 | 0.605 | 17 | 28 | 0.542 |
| Yes | 7 | 8 |  | 7 | 8 |  | 7 | 8 |  |
| Age at onset |  |  |  |  |  |  |  |  |  |
| Mean | 2.68 | 2.82 | 0.669 | 3.00 | 2.72 | 0.410 | 2.49 | 2.76 | 0.445 |
| St. Dev | 0.17 | 0.29 |  | 0.82 | 1.72 |  | 1.02 | 1.70 |  |
| Seizure onset |  |  |  |  |  |  |  |  |  |
| Normal | 22 | 12 | 0.580 | 13 | 21 | 0.536 | 13 | 21 | 0.749 |
| Stormy | 15 | 11 |  | 12 | 14 |  | 11 | 15 |  |
| Mortality |  |  |  |  |  |  |  |  |  |
| No | 37 | 22 | 0.201 | 28 | 31 | 0.394 | 24 | 35 | 0.410 |
| Yes | 0 | 1 |  | 0 | 1 |  | 0 | 1 |  |
| Myoclonic-atonic seizures at onset | | | |  |  |  |  |  |  |
| No | 17 | 10 | 0.852 | 16 | 11 | 0.012* | 13 | 14 | 0.243 |
| Yes | 20 | 13 |  | 9 | 24 |  | 11 | 22 |  |
| Tonic-vibratory seizures at onset | | |  |  |  |  |  |  |  |
| No | 18 | 11 | 0.951 | 7 | 22 | 0.008* | 9 | 20 | 0.170 |
| Yes | 19 | 12 |  | 18 | 13 |  | 15 | 16 |  |
| Absence seizures at onset |  |  |  |  |  |  |  |  |  |
| No | 30 | 15 | 0.168 | 19 | 26 | 0.629 | 15 | 30 | 0.067 |
| Yes | 7 | 8 |  | 6 | 6 |  | 9 | 6 |  |
| Myoclonic seizures at onset |  |  |  |  |  |  |  |  |  |
| No | 26 | 19 | 0.283 | 18 | 27 | 0.650 | 18 | 27 | 0.175 |
| Yes | 11 | 4 |  | 7 | 8 |  | 9 | 9 |  |
| Genetic Test |  |  |  |  |  |  |  |  |  |
| Negative | 17 | 6 | 0.367 | 15 | 8 | 0.006* | 16 | 7 | 0.158 |
| Positive | 9 | 6 |  | 3 | 12 |  | 7 | 8 |  |

ID = intellectual disability. ^a^P-values were calculated by χ^2^ test or t-test, as appropriate. ^EDDI: Early Developmental Delay Index (none = reference category).

^*^*P*<0.050

**Supplementary Table 4 .** **Log-rank test performed in a subgroup of 37 patients who achieved seizure freedom.**

| **Variables** | **Observed Event** | | **Expected Event** | ***P-*value** | |  |
| --- | --- | --- | --- | --- | --- | --- |
| Gender |  |  | | |  | |
| F | 7 | 9.28 | | | 0.365 | |
| M | 30 | 27.62 | | |  | |
| Family history of febrile seizures, epilepsy or both | |  | | |  | |
| No | 16 | 17.01 | | | 0.731 | |
| Yes | 19 | 17.99 | | |  | |
| Early language delay |  |  | | |  | |
| No | 26 | 18 | | | 0.008* | |
| Yes | 11 | 19 | | |  | |
| Early motor delay |  |  | | |  | |
| No | 28 | 19.26 | | | 0.004* | |
| Yes | 9 | 17.74 | | |  | |
| EDDI^ | |  | | |  | |
| No | 25 | 16.85 | | | 0.013* | |
| Delay in only one domain (language or motor) | 4 | 3.55 | | |  | |
| Global delay (both language and motor) | 8 | 16.60 | | |  | |
| Febrile seizures |  |  | | |  | |
| No | 30 | 26.43 | | | 0.188 | |
| Yes | 7 | 10.57 | | |  | |
| Stormy onset |  |  | | |  | |
| No | 22 | 22.26 | | | 0.930 | |
| Yes | 15 | 14.74 | | |  | |
| Myoclonic-atonic seizures at onset | |  | | |  | |
| No | 17 | 15.87 | | | 0.706 | |
| Yes | 20 | 21.13 | | |  | |
| Tonic-vibratory seizures |  |  | | |  | |
| No | 18 | 19.80 | | | 0.550 | |
| Yes | 19 | 17.20 | | |  | |
| Absence seizures at onset |  |  | | |  | |
| No | 30 | 25.33 | | | 0.097 | |
| Yes | 7 | 11.67 | | |  | |
| Myoclonic seizures at onset | |  | | |  | |
| No | 26 | 29.51 | | | 0.144 | |
| Yes | 11 | 7.49 | | |  | |
| Genetic aetiology |  |  | | |  | |
| No | 17 | 10.92 | | | 0.013* | |
| Yes | 9 | 15.08 | | |  | |

^*^*P*<0.050. ^EDDI: Early Developmental Delay Index (none = reference category).

**Supplementary table 5. Population-averaged logistic regression with single predictors, adjusted for age at seizure onset and gender, for long-term cognitive and** **neurodevelopmental outcomes over time.**

|  | **Intellectual disability** |  |  |  |  |  |  |  |  |
| --- | --- | --- | --- | --- | --- | --- | --- | --- | --- |
|  |  |  |  | **Gender** | | | **Age at seizure onset** | | |
| **Variables** | **OR (95% CI)** | ***P*** | ***Q*** | **OR (95% CI)** | ***P*** | ***Q*** | **OR (95% CI)** | ***P*** | ***Q*** |
| Family history for epilepsy, febrile seizure or both | 1.441 (0.539–3.7853) | 0.466 | 1.000 | 1.152 (0.395–3.365) | 0.795 | 1.000 | 0.623 (0.401–0.969) | 0.036 | 0.475 |
| Years after seizure onset |  |  |  |  |  |  |  |  |  |
| 1 | 1.343 (0.773–2.334) | 0.294 | 1.000 |  |  |  |  |  |  |
| 2 | 2.611 (1.497–4.556) | <0.001* | <0.001* |  |  |  |  |  |  |
| 5 | 5.603 (3.004–10.312) | <0.001* | <0.001* |  |  |  |  |  |  |
| 10 | 6.509 (3.328–12.731) | <0.001* | <0.001* |  |  |  |  |  |  |
| 15 | 6.403 (3.102–13.212) | <0.001* | <0.001* |  |  |  |  |  |  |
| 20 | 8.926 (3.276–24.319) | <0.001* | <0.001* |  |  |  |  |  |  |
| Early language delay | 20.192 (5.265–77.441) | <0.001* | <0.001* | 0.561 (0.149–2.114) | 0.393 | 1.000 | 1.202 (0.760–1.900) | 0.431 | 1.000 |
| Years after seizure onset |  |  |  |  |  |  |  |  |  |
| 1 | 1.442 (0.870–2.391) | 0.156 | 1.000 |  |  |  |  |  |  |
| 2 | 3.434 (1.610–7.325) | 0.001* | 0.013* |  |  |  |  |  |  |
| 5 | 9.014 (3.555–22.858) | <0.001* | <0.001* |  |  |  |  |  |  |
| 10 | 11.060 (3.993–30.633) | <0.001* | <0.001* |  |  |  |  |  |  |
| 15 | 11.364 (4.004–32.251) | <0.001* | <0.001* |  |  |  |  |  |  |
| 20 | 20.889 (5.892–74.060) | <0.001* | <0.001* |  |  |  |  |  |  |
| Early motor delay | 11.365 (3.116–41.447) | <0.001* | <0.001* | 0.597 (0.176–2.028) | 0.409 | 1.000 | 1.074 (0.695–1.659) | 0.747 | 1.000 |
| Years after seizure onset |  |  |  |  |  |  |  |  |  |
| 1 | 1.402 (0.870–2.258) | 0.165 | 1.000 |  |  |  |  |  |  |
| 2 | 3.094 (1.546–6.192) | 0.001* | 0.013 |  |  |  |  |  |  |
| 5 | 8.089 (3.628–18.035) | <0.001* | <0.001* |  |  |  |  |  |  |
| 10 | 10.286 (4.342–24.368) | <0.001* | <0.001* |  |  |  |  |  |  |
| 15 | 10.875 (4.612–25.639) | <0.001* | <0.001* |  |  |  |  |  |  |
| 20 | 17.642 (5.194–59.921) | <0.001* | <0.001* |  |  |  |  |  |  |
| EDDI^ Delay in only one domain | 6.251 (1.331–29.344) | 0.020* | 0.292 | 0.427 (0.135–1.341) | 0.145 | 1.000 | 1.440 (0.853–2.432) | 0.171 | 1.000 |
| Global delay | 49.711(10.424–237.069) | <0.001* | <0.001* |  |  |  |  |  |  |
| Years after seizure onset |  |  |  |  |  |  |  |  |  |
| 1 | 1.485 (0.737–2.990) | 0.269 | 1.000 |  |  |  |  |  |  |
| 2 | 3.731 (1.779–7.827) | <0.001* | <0.001* |  |  |  |  |  |  |
| 5 | 10.253 (4.453–23.608) | <0.001* | <0.001* |  |  |  |  |  |  |
| 10 | 12.578 (5.096–31.043) | <0.001* | <0.001* |  |  |  |  |  |  |
| 15 | 13.039 (4.947–34.368) | <0.001* | <0.001* |  |  |  |  |  |  |
| 20 | 24.381 (6.466–91.937) | <0.001* | <0.001* |  |  |  |  |  |  |
| Febrile seizures | 1.147 (0.378–3.477) | 0.809 | 1.000 | 0.977 (0.348–2.738) | 0.964 | 1.000 | 0.642 (0.351–1.176) | 0.151 | 1.000 |
| Years after seizure onset |  |  |  |  |  |  |  |  |  |
| 1 | 1.321 (0.886–1.970) | 0.172 | 1.000 |  |  |  |  |  |  |
| 2 | 2.497 (1.388–4.490) | 0.002* | 0.033 |  |  |  |  |  |  |
| 5 | 5.724 (2.816–11.636) | <0.001* | <0.001* |  |  |  |  |  |  |
| 10 | 6.949 (3.176–15.205) | <0.001* | <0.001* |  |  |  |  |  |  |
| 15 | 6.943 (3.062–15.741) | <0.001* | <0.001* |  |  |  |  |  |  |
| 20 | 10.164 (3.263–31.660) | <0.001* | <0.001* |  |  |  |  |  |  |
| Stormy onset | 0.730 (0.271–1.967) | 0.533 | 1.000 | 0.928 (0.331–2.603) | 0.887 | 1.000 | 0.665 (0.362–1.220) | 0.187 | 1.000 |
| Years after seizure onset |  |  |  |  |  |  |  |  |  |
| 1 | 1.324 (0.887–1.975) | 0.169 | 1.000 |  |  |  |  |  |  |
| 2 | 2.517 (1.402–4.518) | 0.002* | 0.033 |  |  |  |  |  |  |
| 5 | 5.820 (2.889–11.724) | <0.001* | <0.001* |  |  |  |  |  |  |
| 10 | 7.057 (3.281–15.181) | <0.001* | <0.001* |  |  |  |  |  |  |
| 15 | 7.049 (3.147–15.789) | <0.001* | <0.001* |  |  |  |  |  |  |
| 20 | 10.246 (3.332–31.510) | <0.001* | <0.001* |  |  |  |  |  |  |
| Myoclonic-Atonic seizure at onset | 10.886 (1.855–63.878) | 0.008* | 0.124 | 0.303 (0.049–1.885) | 0.201 | 1.000 | 0.433 (0.165–1.138) | 0.09 | 1.000 |
| Years after seizure onset |  |  |  |  |  |  |  |  |  |
| 1 | 1.422 (0.798–2.533) | 0.232 | 1.000 |  |  |  |  |  |  |
| 2 | 3.131 (1.326–7.391) | 0.009 | 0.137 |  |  |  |  |  |  |
| 5 | 8.936 (2.953–27.046) | <0.001* | <0.001* |  |  |  |  |  |  |
| 10 | 10.662 (3.044–37.343) | <0.001* | <0.001* |  |  |  |  |  |  |
| 15 | 10.470 (2.883–38.019) | <0.001* | <0.001* |  |  |  |  |  |  |
| 20 | 14.048 (3.150–62.649) | 0.001* | 0.017 |  |  |  |  |  |  |
| Tonic-vibratory seizure at onset | 0.241 (0.075–0.768) | 0.016* | 0.237 | 0.454 (0.136–1.523) | 0.201 | 1.000 | 0.666 (0.383–1.160) | 0.151 | 1.000 |
| Years after seizure onset |  |  |  |  |  |  |  |  |  |
| 1 | 1.363 (0.855–2.172) | 0.193 | 1.000 |  |  |  |  |  |  |
| 2 | 2.792 (1.422–5.484) | 0.003* | 0.048 |  |  |  |  |  |  |
| 5 | 6.676 (3.000–14.859) | <0.001* | <0.001* |  |  |  |  |  |  |
| 10 | 7.836 (3.299–18.618) | <0.001* | <0.001* |  |  |  |  |  |  |
| 15 | 7.735 (3.072–19.472) | <0.001* | <0.001* |  |  |  |  |  |  |
| 20 | 10.882 (3.203–36.976) | <0.001* | <0.001* |  |  |  |  |  |  |
| Absences seizure at onset | 1.031 (0.355–2.996) | 0.956 | 1.000 | 0.942 (0.341–2.606) | 0.909 | 1.000 | 0.652 (0.357–1.192) | 0.165 | 1.000 |
| Years after seizure onset |  |  |  |  |  |  |  |  |  |
| 1 | 1.321 (0.887–1.967) | 0.171 | 1.000 |  |  |  |  |  |  |
| 2 | 2.495 (1.392–4.473) | 0.002* | 0.033 |  |  |  |  |  |  |
| 5 | 5.710 (2.833–11.510) | <0.001* | <0.001* |  |  |  |  |  |  |
| 10 | 6.942 (3.205–15.034) | <0.001* | <0.001* |  |  |  |  |  |  |
| 15 | 6.934 (3.082–15.600) | <0.001* | <0.001* |  |  |  |  |  |  |
| 20 | 10.094 (3.282–31.047) | <0.001* | <0.001* |  |  |  |  |  |  |
| Myoclonic seizure at onset | 0.489 (0.181–1.321) | 0.158 | 1.000 | 0.974 (0.343–2.760) | 0.960 | 1.000 | 0.616 (0.336-1.129) | 0.117 | 1.000 |
| Years after seizure onset |  |  |  |  |  |  |  |  |  |
| 1 | 1.328 (0.888–1.986) | 0.167 | 1.000 |  |  |  |  |  |  |
| 2 | 2.524 (1.396–4.566) | 0.002* | 0.033 |  |  |  |  |  |  |
| 5 | 5.683 (2.761–11.697) | <0.001* | <0.001* |  |  |  |  |  |  |
| 10 | 6.952 (3.081–15.686) | <0.001* | <0.001* |  |  |  |  |  |  |
| 15 | 6.982 (2.946–16.546) | <0.001* | <0.001* |  |  |  |  |  |  |
| 20 | 10.449 (3.134–34.844) | <0.001* | <0.001* |  |  |  |  |  |  |
| Genetic aetiology | 11.913 (2.942–48.250) | 0.001* | 0.017 | 1.880 (0.528–6.700) | 0.330 | 1.000 | 0.972 (0.614–1.541) | 0.614 | 1.000 |
| Years after seizure onset |  |  |  |  |  |  |  |  |  |
| 1 | 1.434 (0.580–3.542) | 0.434 | 1.000 |  |  |  |  |  |  |
| 2 | 3.392 (1.355–8.488) | 0.009* | 0.137 |  |  |  |  |  |  |
| 5 | 13.604 (4.559–40.595) | <0.001* | <0.001* |  |  |  |  |  |  |
| 10 | 11.902 (3.294–43.003) | <0.001* | <0.001* |  |  |  |  |  |  |
| 15 | 11.037 (2.576–47.273) | <0.001* | <0.001* |  |  |  |  |  |  |
| 20 | 20.827 (1.526–284.090) | 0.023 | 0.331 |  |  |  |  |  |  |
| Drug resistance | 5.022 (1.593–15.833) | 0.006* | 0.095 | 2.299 (0.707–7.476) | 0.166 | 1.000 | 0.645 (0.378–1.100) | 0.108 | 1.000 |
| Years after seizure onset |  |  |  |  |  |  |  |  |  |
| 1 | 1.379 (0.887–2.144) | 0.154 | 1.000 |  |  |  |  |  |  |
| 2 | 2.823 (1.482–5.376) | 0.002* | 0.033 |  |  |  |  |  |  |
| 5 | 7.032 (3.343–14.792) | <0.001* | <0.001* |  |  |  |  |  |  |
| 10 | 8.900 (3.988–19.862) | <0.001* | <0.001* |  |  |  |  |  |  |
| 15 | 8.588 (3.644–20.239) | <0.001* | <0.001* |  |  |  |  |  |  |
| 20 | 14.945 (4.329–51.596) | <0.001* | <0.001* |  |  |  |  |  |  |
| **Other developmental comorbidities** | |  |  |  |  |  |  |  |  |
|  |  |  |  |  |  |  |  |  |  |
| Family history for epilepsy, febrile seizure or both | 1.014 (0.390–2.635) | 0.978 | 1.000 | 2.603 (0.866–7.826) | 0.089 | 1.000 | 1.181 (0.805–1.732) | 0.394 | 1.000 |
| Years after seizure onset |  |  |  |  |  |  |  |  |  |
| 1 | 1.730 (0.880–3.401) | 0.112 | 1.000 |  |  |  |  |  |  |
| 2 | 4.315 (2.212–8.418) | <0.001* | <0.001* |  |  |  |  |  |  |
| 5 | 13.372 (6.455–27.698) | <0.001* | <0.001* |  |  |  |  |  |  |
| 10 | 10.594 (4.948–22.238) | <0.001* | <0.001* |  |  |  |  |  |  |
| 15 | 11.237 (5.003–25.239) | <0.001* | <0.001* |  |  |  |  |  |  |
| 20 | 16.445 (5.785–46.747) | <0.001* | <0.001* |  |  |  |  |  |  |
| Early language delay | 3.949 (1.187–13.142) | 0.025* | 0.269 | 1.672 (0.435–6.429) | 0.454 | 1.000 | 1.648 (1.035–2.623) | 0.035 | 0.357 |
| Years after seizure onset |  |  |  |  |  |  |  |  |  |
| 1 | 1.719 (1.085–2.726) | 0.021 | 0.232 |  |  |  |  |  |  |
| 2 | 4.263 (2.202–8.253) | <0.001* | <0.001* |  |  |  |  |  |  |
| 5 | 13.702 (5.736–32.729) | <0.001* | <0.001* |  |  |  |  |  |  |
| 10 | 11.272 (4.684–27.126) | <0.001* | <0.001* |  |  |  |  |  |  |
| 15 | 12.126 (5.018–29.301) | <0.001* | <0.001* |  |  |  |  |  |  |
| 20 | 19.529 (5.824–65.489) | <0.001* | <0.001* |  |  |  |  |  |  |
| Early motor delay | 2.600 (0.804–8.408) | 0.110 | 0.998 | 2.030 (0.531–7.768) | 0.301 | 1.000 | 1.470 (0.948–2.279) | 0.086 | 0.813 |
| Years after seizure onset |  |  |  |  |  |  |  |  |  |
| 1 | 1.701 (1.057–2.735) | 0.029 | 0.302 |  |  |  |  |  |  |
| 2 | 4.150 (2.109–8.164) | <0.001* | <0.001* |  |  |  |  |  |  |
| 5 | 13.231 (5.448–32.134) | <0.001* | <0.001* |  |  |  |  |  |  |
| 10 | 11.168 (4.664–26.743) | <0.001* | <0.001* |  |  |  |  |  |  |
| 15 | 12.027 (4.959–29.168) | <0.001* | <0.001* |  |  |  |  |  |  |
| 20 | 18.709 (5.435–64.403) | <0.001* | <0.001* |  |  |  |  |  |  |
| EDDI^ Delay in only one domain | 3.401 (0.762–15.176) | 0.109 | 1.000 | 1.450 (0.481–4.363) | 0.508 | 1.000 | 1.600 (1.013–2.529) | 0.044 | 0.566 |
| Global delay | 4.517 (1.331–15.327) | 0.016* | 0.237 |  |  |  |  |  |  |
| Years after seizure onset |  |  |  |  |  |  |  |  |  |
| 1 | 1.738 (0.845–3.575) | 0.133 | 1.000 |  |  |  |  |  |  |
| 2 | 4.336 (2.125–8.849) | <0.001* | <0.001* |  |  |  |  |  |  |
| 5 | 14.054 (6.535–30.225) | <0.001* | <0.001* |  |  |  |  |  |  |
| 10 | 11.520 (5.153–25.753) | <0.001* | <0.001* |  |  |  |  |  |  |
| 15 | 12.410 (5.291–29.105) | <0.001* | <0.001* |  |  |  |  |  |  |
| 20 | 20.033 (6.627–60.554) | <0.001* | <0.001* |  |  |  |  |  |  |
| Febrile seizures | 0.685 (0.186–2.528) | 0.570 | 1.000 | 2.339 (0.614–8.919) | 0.213 | 1.000 | 1.197 (0.758–1.888) | 0.441 | 1.000 |
| Years after seizure onset |  |  |  |  |  |  |  |  |  |
| 1 | 1.651 (1.053–2.588) | 0.029* | 0.396 |  |  |  |  |  |  |
| 2 | 3.908 (2.040–7.487) | <0.001* | <0.001* |  |  |  |  |  |  |
| 5 | 12.490 (5.275–29.573) | <0.001* | <0.001* |  |  |  |  |  |  |
| 10 | 10.699 (4.535–25.238) | <0.001* | <0.001* |  |  |  |  |  |  |
| 15 | 11.416 (4.635–28.120) | <0.001* | <0.001* |  |  |  |  |  |  |
| 20 | 17.606 (4.764–65.066) | <0.001* | <0.001* |  |  |  |  |  |  |
| Stormy onset | 0.865 (0.289–2.588) | 0.796 | 1.000 | 2.491 (0.639–9.712) | 0.189 | 1.000 | 1.246 (0.782–1.983) | 0.355 | 1.000 |
| Years after seizure onset |  |  |  |  |  |  |  |  |  |
| 1 | 1.647 (1.054–2.574) | 0.028* | 0.390 |  |  |  |  |  |  |
| 2 | 3.898 (2.065–7.360) | <0.001* | <0.001* |  |  |  |  |  |  |
| 5 | 12.396 (5.294–29.024) | <0.001* | <0.001* |  |  |  |  |  |  |
| 10 | 10.514 (4.538–24.356) | <0.001* | <0.001* |  |  |  |  |  |  |
| 15 | 11.203 (4.636–27.070) | <0.001* | <0.001* |  |  |  |  |  |  |
| 20 | 16.896 (4.852–58.834) | <0.001* | <0.001* |  |  |  |  |  |  |
| Myoclonic-Atonic seizure at onset | 1.358 (0.454–4.063) | 0.584 | 1.000 | 2.238 (0.589–8.509) | 0.237 | 1.000 | 1.196 (0.754–1.899) | 0.446 | 1.000 |
| Years after seizure onset |  |  |  |  |  |  |  |  |  |
| 1 | 1.669 (1.044–2.668) | 0.032 | 0.431 |  |  |  |  |  |  |
| 2 | 3.977 (2.055–7.698) | <0.001* | <0.001* |  |  |  |  |  |  |
| 5 | 12.604 (5.375–29.553) | <0.001* | <0.001* |  |  |  |  |  |  |
| 10 | 10.612 (4.576–24.607) | <0.001* | <0.001* |  |  |  |  |  |  |
| 15 | 11.461 (4.764–27.571) | <0.001* | <0.001* |  |  |  |  |  |  |
| 20 | 17.427 (5.274–57.584) | <0.001* | <0.001* |  |  |  |  |  |  |
| Tonic-vibratory seizure at onset | a |  |  | a |  |  | a |  |  |
| Years after seizure onset | a |  |  |  |  |  |  |  |  |
| 1 | a |  |  |  |  |  |  |  |  |
| 2 | a |  |  |  |  |  |  |  |  |
| 5 | a |  |  |  |  |  |  |  |  |
| 10 | a |  |  |  |  |  |  |  |  |
| 15 | a |  |  |  |  |  |  |  |  |
| 20 | a |  |  |  |  |  |  |  |  |
| Absences seizure at onset | 0.435 (0.109–1.734) | 0.238 | 1.000 | 2.275 (0.608–8.515) | 0.222 | 1.000 | 1.174 (0.738–1.866) | 0.499 | 1.000 |
| Years after seizure onset |  |  |  |  |  |  |  |  |  |
| 1 | 1.667 (1.052–2.642) | 0.030 | 0.406 |  |  |  |  |  |  |
| 2 | 3.989 (2.088–7.623) | <0.001* | <0.001* |  |  |  |  |  |  |
| 5 | 12.863 (5.350–30.926) | <0.001* | <0.001* |  |  |  |  |  |  |
| 10 | 10.926 (4.477–26.661) | <0.001* | <0.001* |  |  |  |  |  |  |
| 15 | 11.835 (4.747–29.509) | <0.001* | <0.001* |  |  |  |  |  |  |
| 20 | 17.576 (4.917–62.820) | <0.001* | <0.001* |  |  |  |  |  |  |
| Myoclonic seizure at onset | 1.023 (0.359–2.915) | 0.966 | 1.000 | 2.420 (0.639–9.167) | 0.193 | 1.000 | 1.207 (0.758–1.923) | 0.427 | 1.000 |
| Age at seizure onset | 1.207 (0.758–1.923) | 0.427 | 1.000 |  |  |  |  |  |  |
| Years after seizure onset |  |  |  |  |  |  |  |  |  |
| 1 | 1.655 (1.052–2.603) | 0.029* | 0.396 |  |  |  |  |  |  |
| 2 | 3.919 (2.056–7.471) | <0.001* | <0.001* |  |  |  |  |  |  |
| 5 | 12.407 (5.290–29.100) | <0.001* | <0.001* |  |  |  |  |  |  |
| 10 | 10.540 (4.534–24.503) | <0.001* | <0.001* |  |  |  |  |  |  |
| 15 | 11.291 (4.678–27.253) | <0.001* | <0.001* |  |  |  |  |  |  |
| 20 | 17.059 (4.928–59.047) | <0.001* | <0.001* |  |  |  |  |  |  |
| Genetic aetiology | 1.695 (0.477–6.027) | 0.414 | 1.000 | 1.752 (0.512–5.990) | 0.371 | 1.000 | 1.266 (0.786–2.040) | 0.332 | 1.000 |
| Years after seizure onset |  |  |  |  |  |  |  |  |  |
| 1 | 2.334 (0.913–5.965) | 0.076 | 0.925 |  |  |  |  |  |  |
| 2 | 6.157 (2.426–15.624) | <0.001* | <0.001* |  |  |  |  |  |  |
| 5 | 19.950 (7.219–55.138) | <0.001* | <0.001* |  |  |  |  |  |  |
| 10 | 16.021 (5.297–48.454) | <0.001* | <0.001* |  |  |  |  |  |  |
| 15 | 12.937 (3.853–43.447) | <0.001* | <0.001* |  |  |  |  |  |  |
| 20 | 16.950 (2.808–102.333) | 0.002* | 0.033 |  |  |  |  |  |  |
| Drug resistance | 2.191 (0.715–6.707) | 0.170 | 1.000 | 3.254 (0.901–11.758) | 0.072 | 0.882 | 1.234 (0.790–1.929) | 0.356 | 1.000 |
| Years after seizure onset |  |  |  |  |  |  |  |  |  |
| 1 | 1.709 (1.064–2.744) | 0.027* | 0.378 |  |  |  |  |  |  |
| 2 | 4.202 (2.130–8.291) | <0.001* | <0.001* |  |  |  |  |  |  |
| 5 | 13.482 (5.448–33.365) | <0.001* | <0.001* |  |  |  |  |  |  |
| 10 | 11.481 (4.723–27.908) | <0.001* | <0.001* |  |  |  |  |  |  |
| 15 | 12.060 (4.934–29.479) | <0.001* | <0.001* |  |  |  |  |  |  |
| 20 | 18.452 (5.448–62.504) | <0.001* | <0.001* |  |  |  |  |  |  |

**P*<0.050; * FDR-adjusted Q< 0.050; ^EDDI: Early Developmental Delay Index (none = reference category).

**Supplementary table 6.** **Multivariate population–averaged logistic regression evaluating significant predictors of intellectual disability and other neurodevelopmental comorbidities over time**

| **Intellectual disability** |  |  |  |  |  |  |  |  |  |
| --- | --- | --- | --- | --- | --- | --- | --- | --- | --- |
|  |  |  |  | **Gender** | | | **Age at seizure onset** | | |
| **Variables** | **OR (95% CI)** | ***P*** | ***Q*** | **OR (95% CI)** | ***P*** | ***Q*** | **OR (95% CI)** | ***P*** | ***Q*** |
| Myoclonic-Atonic seizure at onset | 5.348 (1.866–15.323) | 0.002* | 1.000 | 0.287 (0.084–0.984) | 0.047 | 0.601 | 1.482 (0.844–2.603) | 0.171 | 1.000 |
| EDDI^ Delay in only one domain | 3.939 (0.779–19.918) | 0.097 | 1.000 |  |  |  |  |  |  |
| Global delay | 54.438 (10.296–287.817) | <0.001* | <0.001* |  |  |  |  |  |  |
| Years after seizure onset |  |  |  |  |  |  |  |  |  |
| 1 | 1.552 (0.713–3.378) | 0.268 | 1.000 |  |  |  |  |  |  |
| 2 | 4.257 (1.890–9.588) | <0.001* | <0.001* |  |  |  |  |  |  |
| 5 | 12.827 (5.174–31.801) | <0.001* | <0.001* |  |  |  |  |  |  |
| 10 | 16.465 (6.101–44.436) | <0.001* | <0.001* |  |  |  |  |  |  |
| 15 | 17.873 (6.140–52.026) | <0.001* | <0.001* |  |  |  |  |  |  |
| 20 | 32.841 (7.992–134.951) | <0.001* | <0.001* |  |  |  |  |  |  |
| Myoclonic-Atonic seizure at onset | 4.711 (1.685–13.173) | 0.003* | 0.048* | 0.268 (0.076–0.937) | 0.039 | 0.508 | 1.385 (0.806–2.379) | 0.238 | 1.000 |
| EDDI^ Delay in only one domain | 3.805 (0.809–17.900) | 0.091 | 1.000 |  |  |  |  |  |  |
| Global delay | 31.984 (6.769–151.117) | <0.001* | <0.001* |  |  |  |  |  |  |
| Time | 1.193 (1.133–1.257) | <0.001* | <0.001* |  |  |  |  |  |  |
| **Other developmental comorbidities** | |  |  |  |  |  |  |  |  |
|  | |  |  |  |  |  |  |  |  |
| Myoclonic-Atonic seizure at onset |  |  |  | 1.450 (0.481–4.363) | 0.508 | 1.000 | 1.600 (1.013–2.529) | 0.044 | 0.566 |
| EDDI^ Delay in only one domain | 3.401 (0.762–15.176) | 0.109 | 1.000 |  |  |  |  |  |  |
| Global delay | 4.517 (1.331–15.327) | 0.016* | 0.237 |  |  |  |  |  |  |
| Years after seizure onset |  |  |  |  |  |  |  |  |  |
| 1 | 1.738 (0.845–3.575) | 0.133 | 1.000 |  |  |  |  |  |  |
| 2 | 4.336 (2.125–8.849) | <0.001* | <0.001* |  |  |  |  |  |  |
| 5 | 14.054 (6.535–30.225) | <0.001* | <0.001* |  |  |  |  |  |  |
| 10 | 11.520 (5.153–24.753) | <0.001* | <0.001* |  |  |  |  |  |  |
| 15 | 12.410 (5.291–29.105) | <0.001* | <0.001* |  |  |  |  |  |  |
| 20 | 20.033 (6.627–60.554) | <0.001* | <0.001* |  |  |  |  |  |  |
| Myoclonic-Atonic seizure at onset |  |  |  | 1.523 (0.511–4.538) | 0.875 | 1.000 | 1.497 (0.966–2.321) | 0.071 | 0.875 |
| EDDI^ Delay in only one domain | 3.034 (0.768–11.979) | 0.113 | 1.000 |  |  |  |  |  |  |
| Global delay | 3.278 (1.024–10.492) | 0.045* | 0.582 |  |  |  |  |  |  |
| Time | 1.145 (1.101–1.191) | <0.001* | <0.001* |  |  |  |  |  |  |

**P*<0.050; * FDR-adjusted Q< 0.050; ^EDDI: Early Developmental Delay Index (none = reference category).

**Supplementary references**

1. Buijsse N, Jansen FE, Ockeloen CW, et al. Epilepsy is an important feature of KBG syndrome associated with poorer developmental outcome. Epilepsia Open 2023;8(4):1300-1313.

2. Alves RM, Uva P, Veiga MF, et al. Novel ANKRD11 gene mutation in an individual with a mild phenotype of KBG syndrome associated to a GEFS+ phenotypic spectrum: a case report. BMC Medical Genetics 2019;20(1):16.

3. Auconi M, Serino D, Digilio MC, et al. Epilepsy in KBG syndrome. Developmental Medicine & Child Neurology 2023;65(5):712-720.

4. Whitney R, Komar M, Yoganathan S, et al. Epilepsy in KBG Syndrome: Report of Additional Cases. Pediatric Neurology 2024;151:138-142.

5. Carvill GL, Heavin SB, Yendle, SC, et al. Targeted resequencing in epileptic encephalopathies identifies de novo mutations in CHD2 and SYNGAP1. Nature Genetics 2013;45(7):825–830.

6. Trivisano M, Striano P, Sartorelli J et al. CHD2 mutations are a rare cause of generalized epilepsy with myoclonic-atonic seizures. Epilepsy & Behavior 2015;51:53-56.

7. Chen J, Zhang J, Liu A, et al. CHD2-related epilepsy: novel mutations and new phenotypes. Developmental Medicine & Child Neurology 2020;62(5):647-653.

8. Angione K, Eschbach K, Smith G, et al. Genetic testing in a cohort of patients with potential epilepsy with myoclonic-atonic seizures. Epilepsy Research 2019;150:70-77.

9. Routier L, Verny, F, Barcia G, et al. Exome sequencing findings in 27 patients with myoclonic-atonic epilepsy: Is there a major genetic factor? Clinical Genetics 2019;96(3):254–260.

10. Nickels K, Kossoff EH, Eschbach K, Joshi C. Epilepsy with myoclonic-atonic seizures (Doose syndrome): Clarification of diagnosis and treatment options through a large retrospective multicenter cohort. Epilepsia 2021;62(1):120–127.

11. Ernst ME, Baugh EH, Thomas A, et al. CSNK2B: a broad spectrum of neurodevelopmental disability and epilepsy severity. Epilepsia 2021;62(7), e103-e109.

12. Kim SY, Jang SS, Kim JI, et al. Dissecting the phenotypic and genetic spectrum of early childhood-onset generalized epilepsies. Seizure 2019; 71:222-228.

13. Hinokuma N, Nakashima M, Asai H, et al. (2020) Clinical and genetic characteristics of patients with Doose syndrome. Epilepsia Open 2020;5(3):442-450.

14. Tang S, Addis L, Smith A, et al. Phenotypic and genetic spectrum of epilepsy with myoclonic atonic seizures. Epilepsia 2020;61(5):995–1007.

15. Djordjevic D, Pinard M, Gauthier MS, et al. De novo variants in POLR3B cause ataxia, spasticity, and demyelinating neuropathy. American Journal of Human Genetics 2021;108(1):186-193. Erratum in: American Journal of Human Genetics 2022;109(4):759-763.

16. Symonds JD, Park KL, Mignot C, et al. POLR3B is associated with a developmental and epileptic encephalopathy with myoclonic-atonic seizures and ataxia. Epilepsia 2024;65(11):3303-3323.

17. Tang S, Hughes E, Lascelles K, et al. New SMARCA2 mutation in a patient with Nicolaides–Baraitser syndrome and myoclonic astatic epilepsy. American Journal of Medical Genetics 2020;173A:195–199.

18. Syrbe S, Hedrich UBS, Riesch E, et al. De novo loss- or gain-of-function mutations in KCNA2 cause epileptic encephalopathy. Nature Genetics 2015;47:393–399.

19. Masnada S, Hedrich UBS, Gardella E, et al. Clinical spectrum and genotype-phenotype associations of KCNA2-related encephalopathies. Brain 2017;140(9):2337-2354.

20. Ebach K, Joos H, Doose H, et al. SCN1A mutation analysis in myoclonic astatic epilepsy and severe idiopathic generalized epilepsy of infancy with generalized tonic-clonic seizures. Neuropediatrics 2005;36(3):210-213.

21. Yordanova I, Todorov T, Dimova P, et al. One novel Dravet syndrome causing mutation and one recurrent MAE causing mutation in SCN1A gene. Neuroscience Letters 2011;494(2):180-183.

22. Wolff M, Johannesen KM, Hedrich UBS, et al. Genetic and phenotypic heterogeneity suggest therapeutic implications in SCN2A-related disorders. Brain 2017;140(5):1316–1336.

23. Kim SH, Seo J, Kwon SS, et al. Common genes and recurrent causative variants in 957 Asian patients with pediatric epilepsy. Epilepsia 2024;65(3):766-778.

24. Helbig I, Lopez-Hernandez T, Shor O, et al. A Recurrent Missense Variant in AP2M1 Impairs Clathrin-Mediated Endocytosis and Causes Developmental and Epileptic Encephalopathy. American Journal of Human Genetics 2019;104(6):1060–1072.

25. Stamberger H, Hammer TB, Gardella E, et al. NEXMIF encephalopathy: an X-linked disorder with male and female phenotypic patterns. Genetics in Medicine 2021;23(2):363-373.

26. De Lange IM, Helbig KL, Weckhuysen S, et al. De novo mutations of KIAA2022 in females cause intellectual disability and intractable epilepsy. Journal of Medical Genetics 2016;53:850–858.

27. Schubert J, Siekierska A, Langlois M, et al. Mutations in STX1B, Encoding a presynaptic protein, cause fever-associated epilepsy syndromes. Nature Genetics 2014;46:1327–1332.

28. Burghardt K, Baba N, Schreyer I, et al. STX1B-related epilepsy in a 24-month-old female infant. Epilepsy Behavior Reports 2020;15:100391.

29. Mignot C, von Stulpnagel C, Nava C, et al. Genetic and neurodevelopmental spectrum of SYNGAP1-associated intellectual disability and epilepsy. Journal of Medical Genetics 2016;53:511–522.

30. Berryer MH, Hamdan FF, Klitten LL, et al. Mutations in SYNGAP1 cause intellectual disability, autism, and a specific form of epilepsy by inducing haploinsufficiency. Human Mutation 2013;34(2):385-394.

31. Liu J, Tong L, Song S, et al. Novel and de novo mutations in pediatric refractory epilepsy. Molecular Brain 2018;11(1):48. Erratum in: Molecular Brain 2018;11(1):59.

32. Lo Barco T, Kaminska A, Solazzi R et al. SYNGAP1-DEE: A visual sensitive epilepsy. Clinical Neurophysiology 2021;132(4):841-850.

33. Cordovado A, Schaettin M, Jeanne M, et al. SEMA6B variants cause intellectual disability and alter dendritic spine density and axon guidance. Human Molecular Genetics 2022;31(19):3325–3340.

34. Møller RS, Wuttke TV, Helbig I, et al. Mutations in GABRB3: From febrile seizures to epileptic encephalopathies. Neurology 2017;88(5):483–492.

35. Epi4K Consortium. De Novo Mutations in SLC1A2 and CACNA1A Are Important Causes of Epileptic Encephalopathies. American Journal of Human Genetics 2016;99(2):287-298.

36. Yang Y, Zeng Q, Cheng M, et al. GABRB3-related epilepsy: novel variants, clinical features and therapeutic implications. Journal of Neurology 2022;269(5):2649-2665.

37. Johannesen KM, Gardella E, Linnankivi T et al. Defining the phenotypic spectrum of SLC6A1 mutations. Epilepsia 2018;59(2):389-402.

38. Carvill GL, McMahon JM, Schneider A, et al. Mutations in the GABA Transporter SLC6A1 Cause Epilepsy with Myoclonic-Atonic Seizures. American Journal of Human Genetics 2015;96(5):808–815.

39. Mermer F, Poliquin S, Zhou S, et al. Astrocytic GABA transporter 1 deficit in novel SLC6A1 variants mediated epilepsy: Connected from protein destabilization to seizures in mice and humans. Neurobiology of Disease 2022;172:105810.

40. Silva DB, Trinidad M, Ljungdahl A, et al. Haploinsufficiency underlies the neurodevelopmental consequences of SLC6A1 variants. American Journal of Human Genetics 2024;111(6):1222-1238.

41. Goodspeed K, Pérez-Palma E, Iqbal S, et al. Current knowledge of SLC6A1-related neurodevelopmental disorders. Brain Communications 2020;2(2):fcaa170.

42. Palmer S, Towne MC, Pearl PL, et al. SLC6A1 Mutation and Ketogenic Diet in Epilepsy With Myoclonic-Atonic Seizures. Pediatric Neurology 2016;64:77-79.

43. Zech M, Jech R, Wagner M, et al. Molecular diversity of combined and complex dystonia: insights from diagnostic exome sequencing. Neurogenetics 2017;18(4):195-205.

44. Mattison KA, Butler KM, Inglis GAS, et al. SLC6A1 variants identified in epilepsy patients reduce γ-aminobutyric acid transport. Epilepsia 2018;59(9):e135-e141. Erratum in: Epilepsia 2019;60(8):1751.

45. Yuan H, Wang Q, Li Y, et al. Concurrent pathogenic variants in SLC6A1/NOTCH1/PRIMPOL genes in a Chinese patient with myoclonic-atonic epilepsy, mild aortic valve stenosis and high myopia. BMC Medical Genetics 2020;21(1):93.

46. Panda PK, Mandal S, Gupta SK, et al. Low Glycemic Index Therapy in Drug-Refractory SLC6A1 Gene-Related Myoclonic-Astatic Epilepsy. Indian Journal of Pediatrics 2024;91(9):985.

47. Mullen SA, Marini C, Suls A, et al. Glucose transporter 1 deficiency as a treatable cause of myoclonic astatic epilepsy. Archives of Neurology 2011;68(9):1152–1155.

48. Simard-Tremblay E, Berry P, Owens A, et al. High-fat diets and seizure control in myoclonic-astatic epilepsy: a single center's experience. Seizure 2015;25:184-186.

49. Lebon S, Suarez P, Alija S, et al. When should clinicians search for GLUT1 deficiency syndrome in childhood generalized epilepsies? European Journal of Paediatric Neurology 2015;19(2):170-175.

50. Mori T, Sakamoto M, Tayama T, et al. A case of epilepsy with myoclonic atonic seizures caused by SLC6A1 gene mutation due to balanced chromosomal translocation. Brain Development 2023;45(7):395-400.

51. Vlaskamp DR, Rump P, Callenbach PM, et al. Haploinsufficiency of the STX1B gene is associated with myoclonic astatic epilepsy. European Journal of Paediatric Neurology 2016;20(3):489-492.

52. Balestrini S, Milh M, Castiglioni C, et al. TBC1D24 genotype-phenotype correlation: Epilepsies and other neurologic features. Neurology 2016;87(1):77–85.
